# Polymeric Microneedles for Health Care Monitoring: An Emerging Trend

**DOI:** 10.1021/acssensors.4c00612

**Published:** 2024-04-24

**Authors:** Raquel L. Pereira, K. B. Vinayakumar, Sanna Sillankorva

**Affiliations:** INL − International Iberian Nanotechnology Laboratory, Av. Mestre José Veiga, 4715-330 Braga, Portugal

**Keywords:** microneedle, healthcare, monitoring, biomarkers, analyte, biosensor, interstitial fluid, bioreceptor

## Abstract

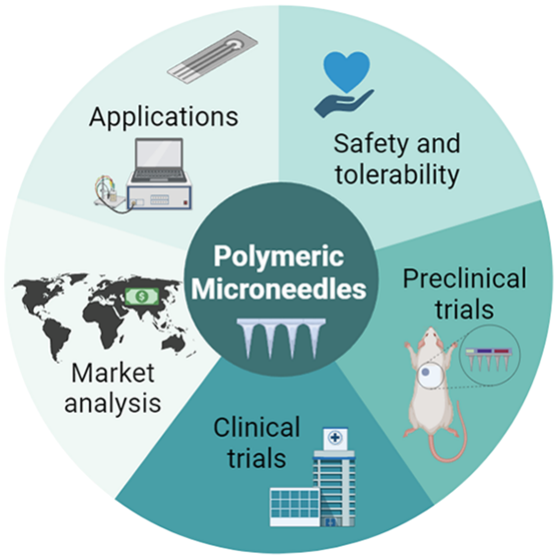

Bioanalyte collection by blood draw is a painful process, prone to needle phobia and injuries. Microneedles can be engineered to penetrate the epidermal skin barrier and collect analytes from the interstitial fluid, arising as a safe, painless, and effective alternative to hypodermic needles. Although there are plenty of reviews on the various types of microneedles and their use as drug delivery systems, there is a lack of systematization on the application of polymeric microneedles for diagnosis. In this review, we focus on the current state of the art of this field, while providing information on safety, preclinical and clinical trials, and market distribution, to outline what we believe will be the future of health monitoring.

Even after 100 years of invention, microneedles (MNs) still attract genuine interest from industries and academics.^1^ The first patent for solid and hollow MNs was filed in 1971.^2^ Following this, in 1973, a patent for drug-coated MNs was filed,^3^ and, in 1998, the concept of silicon microfabricated MNs for drug delivery was reported.^4^ The first reports on the delivery of vaccines, macromolecules, nanoparticles, and genetic materials happened in 2001 and 2002.^5,6^

Traditionally, biomolecules are collected from blood, using painful hypodermic needles, which cause stress/anxiety, and are prone to needle stick injuries.^7,8^ The advancement in MN technology has led to the exploration of dermal interstitial fluid (ISF) as an alternative source of biomarkers. The skin is typically composed of the *stratum corneum*, epidermis, and dermis. Even though the epidermis contains a small amount of ISF, most of the ISF is contained in the dermis.^9^ ISF is composed of peptides, proteins, electrolytes, water, and other nutrients that can be used for continuous health monitoring and diagnostics. Furthermore, the use of ISF in diagnostics is amplified by the large skin area and total volume of ISF available (which is 3 times higher than blood).^7^ To reach the ISF, MNs should have a height of ∼500–900 μm, depending on the skin type, ethnicity, and age of the person. In addition, the diameter/width and the tip diameter of the MNs should be optimized to reduce insertion pain.^10−12^

The use of MN arrays for ISF collection and analysis started with hollow glass MNs to draw glucose. The results obtained compared with blood glucose levels and fell under the clinical acceptance range.^13,14^ Since then, swellable, solid, hydrogel, and sponge-forming MNs have been demonstrated to collect/draw ISF, along with hollow MNs.^14−19^ Broadly the use of MNs for diagnostics can be split into two categories: (i) MNs used to collect biomarkers from the ISF, which will then be analyzed outside the MN array using traditional benchtop methods;^20−22^ (ii) sensing element(s) integrated into the MN arrays and used to detect biomarkers *in situ* using electrochemical or optical approaches.^23−26^

MNs for sensing applications can be produced from a variety of materials, such as silicon, metals or polymers.^27−29^ However, polymeric MNs have been recently attracting attention in the fabrication of monitoring devices because of their enhanced biocompatibility, advantageous mechanical properties, easy, low-cost and reproducible manufacture, ability to quickly draw ISF, and large variety of physicochemical properties.^30−32^

During the last decades, several MN reviews have been published to cover the wide depth and breadth of MN technology in the drug delivery and cosmetics sectors.^33−40^ Different review articles have also been published covering the developments in ISF sampling, MN-based sensors/wearables, and wearable electrochemical sensors.^27,41−48^ Although there are plenty reviews focused on the application of polymeric MNs for drug delivery applications,^49−57^ there are only a few covering the use of polymeric MNs for health monitoring.^30,58^ As such, we devised this review to summarize the work that has been recently published on this topic and provide an outlook on the role of polymeric MNs in diagnostics and its potential for the future of the healthcare market. We start with a discussion of the types of MNs and their fabrication approaches and then present a detailed overview of the recent work published on the use of MNs for diagnostics. In this overview, we cover aspects related to the type of polymers used to fabricate the MNs, the analysis method and the analyte(s) targeted. We later discuss the safety and efficacy of MN-based devices and the main findings collected in preclinical and clinical trials. We further analyze the market distribution and economic importance of MN-based devices. Lastly, we provide a glimpse of the future trends in MNs for diagnosis and highlight the obstacles to overcome to introduce MN-based sensing devices in the clinic.

## Types of Microneedles for Monitoring Approaches and Their Manufacturing

**Figure 1:**
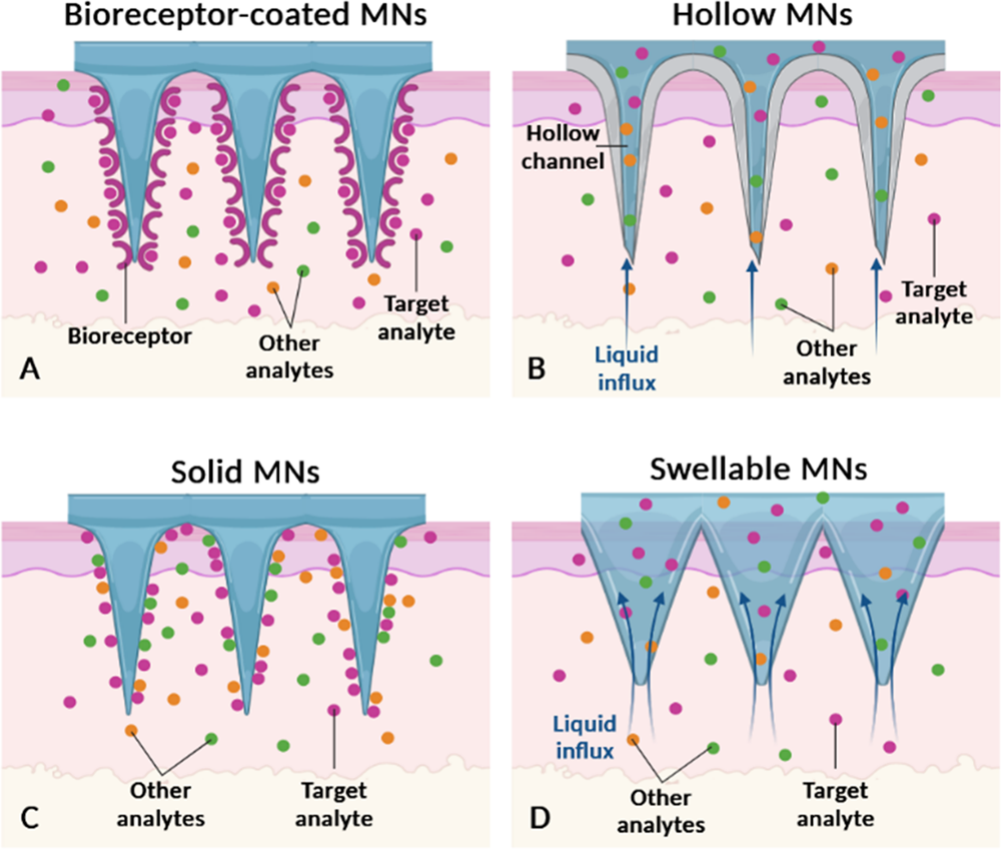
Types of polymeric microneedles and their analyte collection strategies. (A) Microneedles coated with a layer of bioreceptors; (B) hollow microneedles; (C) solid microneedles; and (D) swellable microneedles. Adapted with permission from *Sil et al*.^61^ Copyright 2024 Elsevier.

There are different types of MNs reported in literature, however for monitoring approaches only solid, hollow, coated, and swellable are utilized (Figure 1). These MNs can be made of varied materials, including silicon, metals, biomaterials, and polymers.^27−29^ The choice of MN fabrication process depends on the materials used, critical dimensions required, and functionalities. MN fabrication can be divided based on the type of MN into different categories, each having its advantages and disadvantages. Only a few fabrication processes are referred (for a more thorough reading, please see the review articles^59,60^).

### Solid Microneedles

Solid MNs are the simplest type of MNs, formed by solid micron-sized projections made of only one material. The first solid MNs fabricated for ISF collection contained planar arrays of five MNs and were made using 316 stainless steel. These MNs had a 10-μm diameter tip and their lengths varied from 250 to 650 μm. The developed MNs were used to collect ISF from 21 human participants for ∼20 min, using a vacuum pump for suction. The collected ISF was used to analyze several clinical biomarkers and the results were comparable with typical blood sample analysis.^62,63^ However, due to the complications of vacuum setup and time-consuming procedures, the use of metal MNs for ISF collection has not attained much attention and has shifted to polymers [e.g., polylactic acid (PLA), polyglycolic acid (PGA)] due to their skin compatibility.^15^ Unlike metal MNs, solid polymeric MNs can draw fluid by capillarity, bypassing the need for vacuum pumps.

Solid MNs can be produced from molds, using for example soft lithography, micromolding,^64^ hot embossing^65^ and injection molding^66^ techniques. These are primarily pattern transfer techniques, where the material is applied into existing molds to create MNs. They are ideal for mass production and consistent scalable fabrication. However, they have limited design flexibility, which does not allow the fabrication of complex geometries.

Solid MNs can also be directly created from the substrates using chemical etching,^67^ lithography,^68^ or deep reactive ion etching,^69^ yielding structures that have precise geometry, high-aspect-ratio, and can be scaled for mass production. For these processes, a wide range of substrate materials can be used, including silicon, metals, and polymers. However, these techniques may result in rough surface finishes. In addition, they require specialized equipment, the process is often complex and demands expertise for operation. MNs can also be created using photolithography,^70^ femtosecond laser processing,^71^ and electrical discharge machining (EDM).^72^ These processes create precise, complex, intricate and customized MN designs. However, they once again require specialized equipment and expertise for operation. Moreover, complex designs are time-consuming, and only certain substrates can be used. Finally, 3D printing,^73^ an additive manufacturing technique, has been used to build MNs layer-by-layer from digital designs. This technique is suitable for rapid prototyping and customization of complex geometries from a variety of available resins, but is limited by its resolution (lower compared to traditional MN fabrication techniques), and lower mechanical strength. There are also biocompatibility issues because of the limited medical-grade resins available, and often, there are postprocessing steps that might be needed to achieve the desired surface finish.

### Hollow Microneedles

Hollow MNs are miniaturized versions of hypodermic needles, which use their inner channels to collect fluid. They have been fabricated from a variety of materials, such as metals (e.g., stainless steel, nickel, titanium), polymers (e.g., polydimethylsiloxane, poly(methyl methacrylate)), silicon, and ceramics (silicon carbide, alumina), and used for detecting bioanalytes. For example, silicon microfabricated hollow MNs were designed and fabricated with a height of 250–350 μm, to draw ISF and measure glucose.^17^ In another study, ultrafine stainless steel MNs were purchased and assembled in a 3D-printed structure to extract ISF (up to 16 μL) and analyze transcriptome and proteome signatures.^74^ Even though hollow MNs have been used to prove the concept of ISF collection, the possibility of needle blockage is a concern.

There are different approaches to fabricate hollow MNs, including lithography with etching, EDM), laser processing, and 3D printing, which have been briefly detailed above for the fabricating of solid MNs. Besides these, drawing lithography has been used for the fabrication of hollow MNs.^75^ Drawing lithography deposits material through a fine nozzle onto a substrate, drawing MNs with controllable dimensions and shapes. However, this technique is unsuitable for mass production and has limitations regarding the materials that can be used and the geometries that can be drawn. Metal electroplating^76^ is also used to fabricate hollow metal MNs. This fabrication method involves electroplating a conductive material onto a template, followed by etching of the template material to create the desired MNs.

### Coated Microneedles

MNs with a coating containing an active material are used to deliver a broad range of active materials (e.g., proteins, peptides, small molecules, DNAs, viruses). This type of MNs carries a lower concentration of active material because this is placed only on the surface of the MNs and not on the core. Nonetheless, the active materials are delivered very rapidly to the skin. Coated MNs have been used in biosensing applications, for example, to sample ISF for the detection of illicit drugs (using polydimethylsiloxane (PDMS) MNs coated with ligand-modified gold nanorods)^77^ or to directly monitor glucose levels (using polyimide MNs coated with glucose-detecting enzymes).^78^

Coating of microneedles is commonly performed by dip coating,^79^ spin coating,^80^ and electrodeposition.^28^ These processes create a thin film in the surfaces to impart specific properties or functionalities. This is suitable for a range of functional coatings, the process is straightforward and the achieved MNs have uniform coating thickness and surface coverage. Nonetheless, there is limited thickness control, the optimization of the coating thickness may require several iterations, and not all coating materials are compatible with the fabrication method or the MN substrate.

### Swellable Microneedles

The advancement in polymer engineering, concerning mechanical robustness upon insertion and ability to hold fluid through swelling or capillary action, has been supporting the development of swellable MNs. Swellable MNs expand after contact with fluids, due to the diffusion of water into the matrix of the needle material. Mostly, polymer-based materials are used to develop swellable MNs. For example, gelatin methacryloyl-based MNs have been developed that swelled from 293% to 423% upon contact with the ISF, depending on the concentration of polymer and UV cross-linking time used.^81^ Swellable MNs can usually collect higher amounts of fluid in a shorter period, as exemplified by methacrylated hyaluronic acid (MeHA) MNs, which were able to collect ∼1.4 μL of ISF in ∼1 min and ∼2.3 μL in ∼10 min on mice skin.^31^ MeHA and chondroitin sulfate, were shown to collect ∼1–2 μL of ISF.^31,82^ Hydrogel polymers, such as poly(vinyl alcohol), chitosan, polyvinylpyrrolidone, and gelatin methacryloyl facilitated the collection of ∼3–20 μL of ISF.^83−85^

Swellable MNs are typically fabricated by casting/molding swellable polymers into PDMS master molds with an inverse geometry of the MNs. Casting is followed by a solidification step to form the MNs, which can then be peeled from these molds.^73,86^ Some polymers may further require cross-linking to improve mechanical strength, control the swelling behavior, or enhance MN stability.^87^

### Sensor-Integrated Microneedles

Sensor-integrated MNs need a slightly different manufacturing approach due to the requirements of colorimetric, fluorescent, or electrochemical measurements. For example, it is necessary to ensure that the quantity of biomarker-sensing material in the MNs is smaller than the amount of material that gives mechanical robustness. Likewise, for electrochemical sensors, gold, silver, and platinum electrodes need to be incorporated into the system (generally, by sputtering, metal evaporation, or printing),^28,88−90^ and then connected to the source meter via an ion-conductive interface or matrix.^45^ The incorporation of the sensing material can be done on the backside of the MN array or along the MN tips.^84,91^

## Microneedle Safety and Tolerability

MNs are generally regarded as a safe and less painful alternative to conventional sampling methods because of their ability to penetrate the skin without contacting blood vessels and nerve fibers.^92,93^ The first observation in humans of painless MN insertion was made by *Kaushik et al.*, in 2001.^94^ Since then, various publications have corroborated this observation. *Haq et al*. stated that MN application produced more “pressing” and “heavy” sensations, whereas hypodermic needles triggered more “sharp” and “stabbing” feelings.^95^*Van Damme et al*. found that the “prick–pain” associated with their MN device, MicronJet, was significantly lower than that of intramuscular injections.^96^ The participants involved in a study by *Frew et al*. reported a positive experience with MNs and the majority preferred them over hypodermic needles.^97^

One of the main safety concerns around MNs regards their ability to elicit skin irritation, i.e., a reversible inflammatory reaction of the skin.^98^ However, this concern has been debunked by numerous articles. *Bal et al*. published a study stating that the irritation caused by MNs of different lengths and geometries was minimal and short-lasted, resolving in less than 2 h.^98^*Van Damme et al*. and *Hoesly et al*. reported that the skin reactions caused by their respective MN devices were mild and transient.^96,99^ Moreover, *Hoesly et al*. stated that the reactions were characterized by self-contained, barely perceptible erythema that rapidly disappeared without the need for external intervention.^99^ Likewise, the MNs used in a study by *Rouphael et al*. were well-tolerated, causing only mild pruritus, tenderness, and erythema.^100^

The other main concern surrounding MNs is the possibility of opening a pathway for pathogen entry upon MN removal.^101^ Nevertheless, *Haq et al*. demonstrated that the skin disruption caused by MNs is transient, repairing itself in less than 24 h. They also showed that MNs create smaller channels that heal faster than the ones created by hypodermic needles, which lowers the risk of pathogen exposure.^95^ Additionally, standard disinfection before MN application can further help mitigate this risk.^101^ Lastly, it is worth mentioning that the small dimension of MNs can decrease needle phobia in patients while fighting needle stick injuries in healthcare workers.^92,101^ Overall, MNs can have a significant impact on patient compliance by presenting themselves as a bypass to the pain and fear of injection associated with conventional hypodermic needles.^101,102^

## Current Trends Using Polymeric Microneedles for Monitoring

Various MN-based sensing devices have been produced based on silicon, metallic or polymeric MNs. Silicon and metals are the classical options for MN production and offer several attractive features related to their rigidity and multiple fabrication methods. However, their utility is hampered by their expensive and laborious fabrication processes, which often require multiple processing steps in a clean room environment. In addition, silicon MNs are brittle and carry an added risk of fracturing upon skin insertion. Lately, polymers have been attracting a great deal of attention as alternatives to these conventional materials.^103,104^ The main techniques applied for polymeric MN production (photolithography, replica molding, 3D printing, and micromachining) are inexpensive and less cumbersome compared to the techniques used to fabricate silicon and metal MNs. Moreover, these techniques require shorter processing cycles and are suitable for large-scale production.^59,104^ The low-cost aspect of polymeric MNs becomes even more pronounced when considering that some of their fabrication techniques allow mold reuse^32^ and the raw material used for fabrication, i.e. polymers, is already more affordable than metals or silicon.^104^ In addition, polymers have the advantage of being easily formable and offering a wide material variety,^59^ both natural and synthetic, some of which with enhanced eco-friendly and biocompatibility features.^30,58^ Due to their unique and vast inherent physicochemical properties, polymers are often preferred for applications where specific surface characteristics are required.^105^ Furthermore, polymeric MNs have a lower risk of breaking upon insertion than silicon MNs due to their softer nature which allows a higher needle flexibility and a better adaptation to the curved nature of the skin.^30,45^ The mechanical properties of polymeric MNs can also be tuned by adjusting their concentration, molecular weight, and cross-linking density.^106^ Overall, polymeric MNs are gathering the attention of the scientific community^58^ and are increasingly being used for the fabrication of health monitoring devices because of their biocompatibility, facile and low-cost production, advantageous mechanical properties, and possibility of extracting large amounts of fluid in a short timespan.^30−32^ Thus, we have decided to focus only on polymeric MNs moving forward.

**Figure 2:**
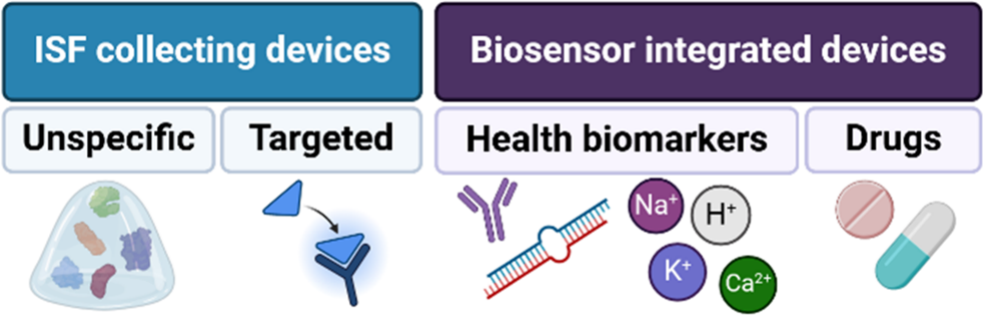
Types of polymeric microneedle-based health monitoring devices.

In this section, we layout the information published in the past 5 years about polymeric MN-based devices for monitoring to provide an overview of the polymers, detection approaches and analytes being explored for health-related applications. To facilitate reading, we have divided them into (i) ISF collecting devices, and (ii) biosensor integrated health monitoring devices (Figure 2).

### ISF Collecting Devices

Polymeric MNs are a minimally invasive, low-cost, and painless alternative to clinically available methods used to collect ISF analytes, such as suction blisters, reverse iontophoresis, and microdialysis.^92,107,108^ In addition, they allow the extraction of large quantities of ISF for analysis, and show improved biocompatibility and mechanical properties.^30−32^ Sample collection devices present in the literature aim to collect biomolecules for posterior benchtop analysis, and can be divided into two categories: (i) those that extract a portion of the whole ISF, which is later processed to separate and analyze specific analytes, and (ii) those that are modified with target-specific moieties (Figure 1A). These bioreceptor-modified MNs have the advantage of capturing exclusively the biomarker of interest, which avoids postprocessing steps, simplifies biodetection and makes this process more efficient.^30,61,109^ Among untargeted MNs, analyte collection can be made through (i) hollow channels (Figure 1B), (ii) nonspecific surface adsorption (Figure 1C), or (iii) swelling of the polymeric matrix (Figure 1D).^61^ In the past 5 years, a total of 9 articles reporting MN-based sampling devices have been published (Table 1). Whole ISF collecting are characterized by the development of MNs that entrap a panoply of bioanalytes via swelling.^108,110−112^ Analyte-specific devices report bioreceptor- modified swellable MNs for the collection of nucleic acids^113^ and articles reporting bioreceptor-modified solid MNs for collecting proteins.^77,109,114,115^ In every case, captured bioanalytes are posteriorly analyzed using standard benchtop techniques, such as immunofluorescence, spectroscopy, or colorimetric assays.

**Table 1:** Summary of the MN-Based Sample Collection Devices Published in the Last 5 Years

Type of MN	MN composition	Method of analysis	Target analyte	Reference
Swellable	Acrylic acid incorporated into gelatin methacrylate	Immunofluorescence (ELISA)	Breast cancer biomarkers	*Huang etal*.^108^
	Porous polydimethylsiloxane (PDMS) coated with hyaluronic acid (HA)	Colorimetry–glucose test paper	Glucose	*Takeuchi etal*.^111^
	Mixture of poly(ethylene glycol) diacrylate and methacrylated hyaluronic acid (MeHA)	Colorimetry or surface-enhanced Raman spectroscopy	Cefazolin, nicotine, paraquat, and methylene blue	*Hsieh etal*.^112^
	Gelatin methacryloyl	UV–vis spectroscopy	Urea	*Fonseca etal*.^110^
Swellable and bioreceptor-modified	Poly-l-lactide coated with alginate	Fluorescence	Cancer-associated RNAs	*Al Sulaiman etal*.^113^
Bioreceptor modified	Mixture of poly(ethylene glycol) diacrylate and poly(ethylene glycol)	Immunofluorescence	Inflammatory cytokines (TNF-α, IL-1β, and IL-6)	*Zhang etal*.^114^
	Polystyrene	Immunofluorescence (p-FLISA)	Pro-inflammatory cytokine (IL-6)	*Wang etal*.^109^
	Polydimethylsiloxane (PDMS)	Surface-enhanced Raman spectroscopy or mass spectroscopy	Fentanyl or alprazolam	*Simas etal*.^77^
	Trimethylolpropane ethoxylate triacrylate	Immunofluorescence or aptamer-based fluorescent quenching	Anemia biomarkers (hemoglobin, ferritin, folic acid, and vitamin B12)	*Wu etal*.^115^

*Huang et al*. cross-linked acrylic acid (AA) with gelatin methacrylate (GelMA) in various proportions to produce GelMA-AA MNs capable of collecting breast cancer biomarkers from the ISF (carcinoembryonic antigen, CEA, and glycoantigen CA15–3). They found that both the GelMA/AA ratio and the cross-linking density play a crucial role in the swelling and mechanical properties of the MN patches. When using an optimal mixture of 1% GelMA + 30% AA, the MNs were able to penetrate the skin without causing nerve damage or pain. Furthermore, they were able to capture CEA and CA15–3 in a mice model, which were later quantified by immunofluorescence (ELISA).^108^

*Takeuchi et al*. produced porous PDMS MNs coated with hyaluronic acid (HA) to draw ISF glucose based on the mechanical compression of the PDMS matrix. By optimizing the porosity ratio, they were able to create MNs (with 60% porosity) that successfully penetrated the skin and extracted ISF at a rate of 0.46 μL/min *in vivo*. Moreover, the successful extraction of glucose was confirmed through the change of color of a glucose test paper placed on the backside of the MNs.^111^

*Hsieh et al*. developed controllable-swelling MNs that, when combined with paper-based colorimetric sensing or surface-enhanced Raman scattering (SERS), provided ultrasensitive biomolecular recognition of cefazolin, nicotine, paraquat, and methylene blue. The MNs, made of a mixture of poly(ethylene gly col) diacrylate and MeHA, could rapidly and reliably extract ISF from the skin. Moreover, in a proof-of-concept in human volunteers, the system effectively detected nicotine levels in ISF, highlighting its potential for personalized medicine.^112^

*Fonseca et al*. fabricated gelatin methacryloyl MNs that were able to collect urea in the ISF (detected through UV–vis spectroscopy). The MNs exhibited high water uptake ability, reaching equilibrium within 20 min, indicating their potential for ISF extraction. MNs showed noncytotoxic behavior toward human keratinocyte cells, confirming their safety for dermal application. Moreover, the MNs successfully penetrated human abdominal skin *ex vivo* and quantified urea from model agarose hydrogels, demonstrating the potential for real-time urea monitoring.^110^

*Al Sulaiman et al*. presented poly-l-lactide MN patches coated with peptide nucleic acid probe-functionalized alginate for multiplex sampling of specific cancer-associated microRNAs from the ISF. The MNs could sample up to 6.5 μL of fluid in 2 min, with a sampling rate cof 0.74 μL/min, outperforming existing sampling technologies. Moreover, they demonstrated high specificity and sensitivity in human skin biopsies, detecting target concentrations as low as 6 nM.^113^

*Zhang et al*. created MNs, made of a mixture of poly(ethylene glycol) diacrylate and poly(ethylene glycol), integrated with photonic crystal barcodes for the noninvasive detection of ISF inflammatory cytokines. The photonic barcodes were decorated with specific probes and enabled biomarker enrichment and detection through immunofluorescence in a sepsis mouse model. These encoded MNs showed advantages over existing MNs for ISF detection, including simplified procedures, multiplex detection capability, and *in vivo* detection preserving the biological activity of targets.^114^

*Wang et al*. introduced a polystyrene MN patch functionalized with biorecognition elements to capture protein biomarkers selectively. The patches exhibited sufficient mechanical strength for skin penetration without yielding, minimal invasiveness, and excellent biocompatibility. Furthermore, when combined with Plasmonic Fluor-Linked Immunosorbent Assay (p-FLISA), they were capable of local biomarker detection with high sensitivity in mice.^109^

*Simas et al*. modified PDMS MNs with ligand-coated gold nanoparticles to detect potent drugs of abuse in the ISF. When combined with SERS or mass spectroscopy, this multimodal detection approach was able to distinguish between fentanyl, alprazolam, or mixtures thereof with high accuracy in human patient samples.^77^

*Wu et al*. functionalized trimethylolpropane ethoxylate triacrylate MNs with antibodies to detect anemia biomarkers. When using immunofluorescence or aptamer-based fluorescent quenching, the MNs demonstrated high specificity and sensitivity and yielded results within 20 min. The sensitivities for ferritin, folic acid, and Vitamin B12 were significantly enhanced compared to conventional methods, achieving lower limits of detection (LODs) achieved. The integrated device maintained sensitivity for up to 21 days of storage without significant degradation, suggesting practical usability and stability.^115^

Overall, the research collectively demonstrates that MNs have great feasibility, efficacy and versatility for minimally invasive ISF sampling, offering potential applications in biomarker monitoring, and disease diagnosis. The articles focused various aspects, including optimization, fabrication techniques, mechanical properties, fluid extraction performance, and sensing capabilities. The key factors influencing the performance and efficacy of MN devices for ISF collection account: 1) composition and fabrication optimization to guarantee controlled swelling performance or ISF extraction, mechanical strength, and skin penetration efficiency; 2) porosity, pore size, and surface coatings play critical roles in fluid extraction rate and biosensing performance; 3) integration with sensing platforms allows for the detection of specific biomarkers, including proteins, nucleic acids, and small molecules, with high sensitivity and selectivity. Techniques such as plasmonic fluorimetry and SERS enable ultrasensitive detection even at low concentrations; 4) *ex vivo* and *in vivo* evaluations confirm successful skin penetration and reliable ISF extraction.

### Targeted Disease and Health Monitoring *in Situ*

**Figure 3:**
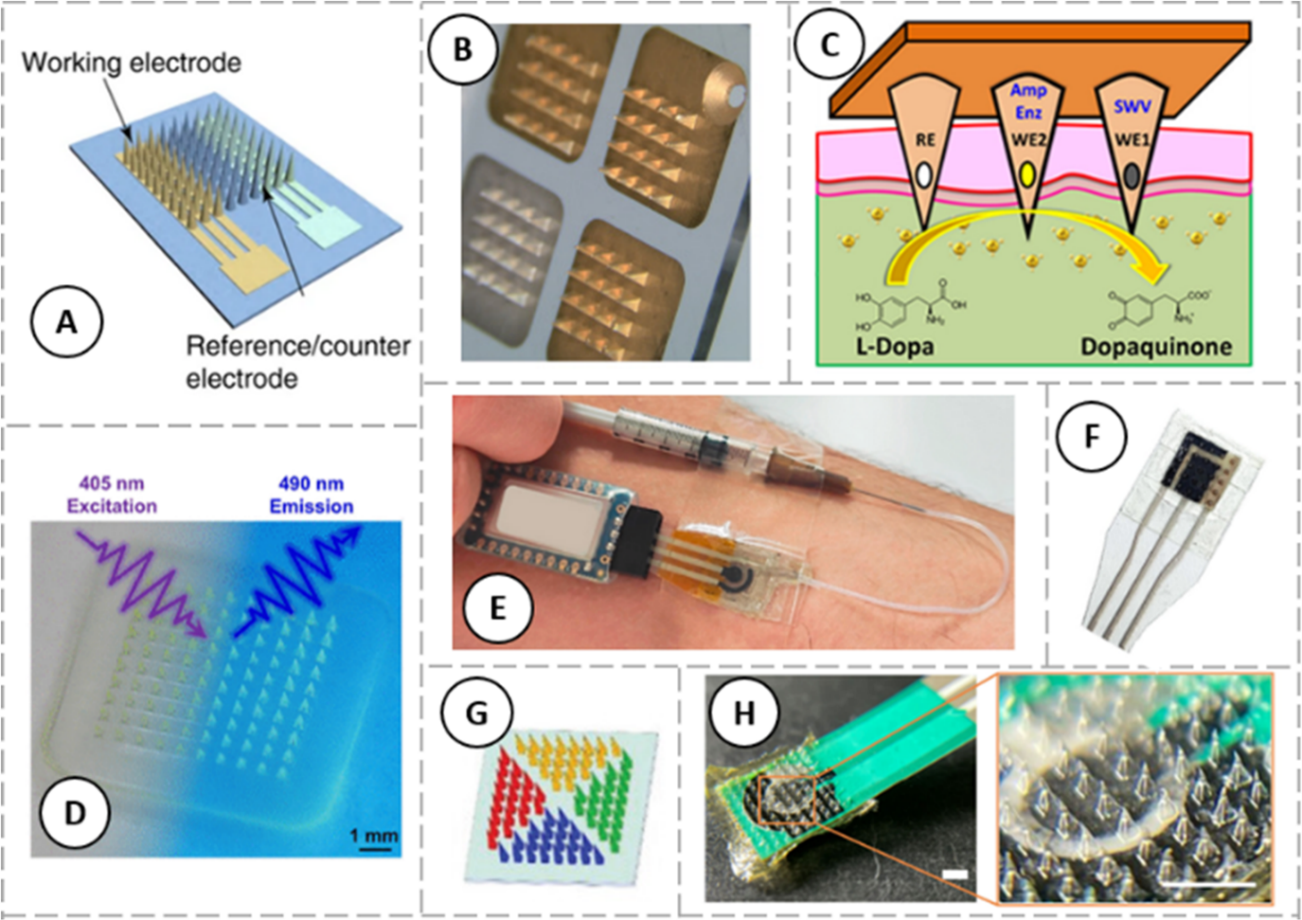
Representative examples of polymeric microneedle-based health monitoring devices. (A) Schematic illustration of a 3D-printed microneedle array produced using clear resin and sputtered with gold to form a conductive 3-electrode system. Adapted from *Liu et al*.;^23^ (B) Microneedle base used to produce a sensor to detect the concentration of antibiotics in interstitial fluid (ISF). Adapted from *Rawson et al*.;^126^ (C) Schematic representation of a microneedle sensor for L-Dopa monitoring in ISF. Adapted with permission from *Goud et al*.^127^ Copyright 2019 American Chemical Society; (D) Image of a fluorescence-based biodegradable microneedle glucose sensor. Adapted from *Sang et al*.;^128^ (E) Image of the wearable glucose sensing patch on a human arm. Adapted with permission from *Parrilla et al*.^124^ Copyright 2022 Elsevier; (F) Single-walled carbon nanotube modified microneedle sensor. Adapted with permission from *Drăgan et al*.^129^ Copyright 2023 Elsevier; (G) Patterned microneedle array for fabricating colorimetric dermal tattoo biosensors. Adapted from *He et al*.;^26^ (H) Digital photo of a microneedle-glucose sensor and zoom-in image of microneedle structure before fluid extraction. Scale bar: 2 mm. Adapted from *Zheng et al*.^93^

Unlike previously mentioned devices, biosensor integrated devices allow precise *in situ* analyte analysis.^61^ These devices can target a wide range of biomolecules and can even be inserted into closed-loop systems, such as the ones used in diabetic patients to simultaneously measure glucose levels and administer the corresponding dose of insulin.^116^ Disease biomarker detecting tools have been used for instance to monitor diabetes progression, nasopharyngeal carcinoma, and chronic kidney disease. Recently published MN-based monitoring devices can be grouped based on their sensing principle into optical and electrochemical sensors.^61^ Electrochemical sensors are more common due to their cheap, easily reproducible and upscalable manufacture. However, they present some limitations, mainly related to their need for external energy sources.^117^Figure 3 contains a few representative examples of both electrochemical and optical polymeric MN-based sensors used for health and disease monitoring. Glucose monitoring plays a significant role in monitoring diseases (e.g., diabetes mellitus^118^ and prediabetes^119^ and gestational diabetes^120^), and health conditions (e.g., insulinoma,^121^ metabolic syndrome,^122^ and Cushing’s syndrome^123^). Among recently published MN-based biosensors, glucose monitoring devices are the most common and the only ones reported to have been incorporated into a closed-loop system. *Parrilla et al*. developed their glucose sensing device based on a polyether ether ketone MN array connected to an enzymatic biosensor. The ISF is actively drawn from the skin using a syringe and immediately contacts with a carbon screen-printed electrode modified with glucose oxidase (GOx) to detect glucose.^124^*Zheng et al*. produced an osmolyte-powered hydrogel MN patch made up of maltose and MeHA to extract the ISF via *in situ* swelling. The extracted ISF then contacts with a gold-based electrochemical biosensor modified with GOx to detect glucose levels^18^*Sharifuzzaman et al*. opted for the direct modification of the surface of polyimide MNs with a glucose-detecting enzyme (glucose dehydrogenase, in this case). The MNs were then connected to a custom 3-electrode system which produced the electrochemical readout of the device.^78^*Liu et al*. 3D-printed a MN array using clear resin, which was sputtered with gold to form a conductive 3-electrode system. This was further modified with GOx to form a system capable of detecting glucose in the ISF.^23^. Similarly, *Barrett et al*. electrodeposited platinum onto the surface of Norland Optical Adhesive NOA68 MNs, which were then modified with GOx to detect glucose.^125^*Dervisevic and Voelcker* opted for OrmoComp photoresist coated with a thin layer of gold to produce MN arrays, which were then modified with GOx to enable glucose detection This electrochemical system had the particularity of possessing microcavities that protect the sensing layer from damage upon insertion/removal.^130^.*Zhao et al*. decided on silk and D-sorbitol MNs modified with GOx and pierced with platinum/silver wires to create a glucose monitoring biosensor.^131^*Luo et al*. developed a system capable of simultaneously detecting glucose and administrating the corresponding dose of insulin. This system is formed by hollow chitosan MNs. The inner layer functions as an insulin injector and the outer layer, where GOx is immobilized, serves as a glucose sensor. The whole system is controlled by an electrochemical electrode and the insulin is delivered with the help of a small electroosmotic pump.^116^

Conversely, *Sang et al.*, *Zeng et al.*, *Li et al.* and *Wu et al.* chose nonelectrochemical approaches for glucose detection. *Sang et al*. went for a fluorescent-based MN sensor. Glucose-responsive fluorescent monomers were added to silk fibroin MNs to produce a patch that, when excited with violet light, turned proportionally blue according to the glucose levels present on the extracted ISF.^128^*Zeng et al*. went for a colorimetric biosensor based on colloidal crystal MNs. Clear resin MNs were infused with glucose-responsive colloidal crystals that proportionally shifted from blue to green upon contact with the glucose present in the ISF.^132^*Li et al*. created swellable MeHA MNs modified with GOx-like gold nanoparticles to detect glucose based on colorimetric changes.^133^*Wu et al*. fabricated hollow MNs and coupled them with a glucose paper strip to produce a colorimetric sensor for glucose detection.^134^

Glucose monitoring has also been performed alongside other bioanalytes through multianalyte MN-based devices. *Dai et al*. developed an electrochemical wearable patch based on MeHA MNs to monitor glucose and lactate levels.^135^*He et al*. reported a colorimetric dermal tattoo biosensor made of HA MNs to monitor glucose, pH, uric acid, and temperature.^26^

Overall, the MN monitoring devices developed for glucose monitoring were capable of extracting ISF containing glucose without causing significant damage or pain. Furthermore, these devices exhibited minimal inflammation after insertion into the skin, ensuring their suitability for long-term use. They showed accurate and continuous monitoring of glucose, offering convenient and rapid monitoring of levels without the need for additional processing steps. The studies have all reported extended linear ranges of glucose detection, suitable for monitoring diabetic patients, along with high sensitivity and selectivity against common interferents. *In vivo* experiments on animal models, including diabetic rabbits and mice, demonstrated the reliability, accuracy, and correlation with conventional blood glucose meters, validating the effectiveness of MN-based glucose monitoring devices. Some of these studies explored multiplexed detection capabilities, enabling simultaneous monitoring of multiple biomarkers (e.g., glucose, insulin, pH, uric acid, and temperature), expanding the potential applications of MN-based biosensors. In addition, one of the papers^124^ showed that a microfluidic setup significantly improved the sensing performance of MNs, enhancing glucose transport and detection efficiency, whereas another article^128^ developed a user-friendly interface, including a smartphone app, which facilitated practical use of MN-based glucose monitoring devices, enhancing accessibility and convenience for users.

To monitor nasopharyngeal carcinoma, *Yang et al*. reported a device to detect Epstein–Barr virus (EBV) cell-free DNA, a newly found biomarker for this disease, based on poly(methyl vinyl ether-*alt*-maleic acid) hydrogel MNs, a reverse iontophoresis Au-carbon nanotube membrane and a flexible electrochemical sensor. The device demonstrated good conductivity and efficient capture of EBV. *In vivo* studies showed that early and accurate detection of nasopharyngeal carcinoma tumors is possible, surpassing other sampling methods.^136^ Likewise, *Zheng et al*. developed a multianalyte electrochemical MN sensor array for diagnosing early chronic kidney disease (CKD). Their study combined HA and MeHA to create MNs for ISF extraction. MeHA lacked strength, so non-cross-linked HA was added for reinforcement. HA-MeHA MNs exhibited improved porosity and mechanical strength, enhancing ISF adsorption. The MN patch featured four electrodes for simultaneous sensing of phosphate, uric acid, creatinine, and urea, and showed sensitivity and specificity in physiological conditions. *In vivo* tests demonstrated multiplexed biomarker detection in ISF, with biomarker levels correlating with CKD progression.^137^ Together, these devices have proven effective for the monitoring of pressing healthcare conditions and we expect that, in the future, similar devices will arise that monitor other disease biomarkers, like C-reactive protein which is overexpressed in several immune-system diseases, including lupus and Crohn’s disease.^138−140^

*In situ* monitoring has also been directed at osmolytes, such as sodium, calcium, and potassium, involved in different electrolyte imbalance disorders (e.g., hypernatremia,^141^ hypercalcemia,^142^ and hypokalemia^143^), and responsible for kidney diseases (acute kidney injury, CKD, and, renal dysfunction).^144−146^*Omar et al*. produced polystyrene MNs coupled with an electrochemical sensor to detect sodium, calcium, potassium, and pH from ISF. This wearable device integrated the sensors, an energy-harvesting system, and an IOT technology. The system harnessed energy from body motion and sunlight (Triboelectric Nanogenerator coupled with a solar cell) charging, the battery necessary for sensing. The four sensors exhibited excellent electrical and sensing performance for each analyte, and demonstrated high sensitivity, selectivity, repeatability, and stability over time. Furthermore, real-data on multiple biomarkers was transmitted to a smartphone app, enhancing the convenience of monitoring.^147^

The monitoring of pH is useful to track metabolic acidosis as changes in pH serve as predictors of the onset of circulatory shock.^148^*Dervisevic et al*. fabricated OrmoComp MNs coated with a thin layer of gold and modified with polyaniline to monitor pH changes in ISF. The working and reference electrodes were fabricated using high-density polymeric MN arrays and a polyethylene naphtholate film. The insulating OrmoComp layer prevented electropolymerization. The sensor exhibited high sensitivity, minimal interference from various ions found in ISF, high reproducibility, accurate monitoring and immediate responses of pH changes in *ex vivo* models.^32^

Alcohol consumption is directly linked to many diseases (e.g., alcohol use disorder and liver diseases) and associated with increased risks of cardiovascular diseases and certain cancers (e.g., liver, colon, and esophagus). Furthermore, alcohol can contribute to the development of mental health disorders (e.g., depression and anxiety). *Zheng et al*. developed swelling MeHA MNs adhered to an electrochemical test strip to detect alcohol. To resolve the stretch incompatibility between the expandable MeHA MNs and the waterproofing electrochemical sensor, a chitosan layer was added between the two substrates. The fabricated MN device was able to extract ISF in less than 1 min and to provide accurate real-time alcohol measurements in an *in vitro* skin model, within a 0–20 mM linearity range. Overall, this work demonstrated the fabrication of a low-cost and convenient MN-based monitoring platform that could easily be adapted to other bioanalytes, like glucose.^93^

Glutathione has been suggested as a biomarker for mitochondrial disease.^149^ Zhao et al. created a colorimetric biosensor for glutathione detection based on swelling MNs, made of a poly(vinyl alcohol) (PVA) and sodium alginate hydrogel. The hydrogel MNs achieved a swellable ratio of 150% and were able to rapidly extract ISF (6.4 mg in 15 min). The device was able to accurately detect glutathione *in vitro* (with a limit of detection of 0.36 μM) and *in vivo* in a rat model. Moreover, the results obtained *in vivo* were comparable to the ones obtained for blood using a commercial kit. This platform represents an alternative to currently available test kits for glutathione, which are expensive and nonreusable,^150^ and we believe it holds great promise for the application to other metabolic diseases, such as hemochromatosis, which is characterized by high levels of ferritin.^151^

MN-based monitoring devices can also be used to detect various substances, from medicines to illicit drugs. *Goud et al*. created two systems to monitor the ISF concentration of medications for treating Parkinson’s disease, one for levodopa and one for apomorphine. For the levodopa sensor, three Eshell 200 acrylate-based hollow MNs were produced and filled with unmodified carbon paste, tyrosinase-containing carbon paste, or a silver wire. Electrical contacts were then established to obtain an enzymatic and nonenzymatic sensor capable of detecting levodopa *in vitro* and *ex vivo* (in mice skin), within a linear detection range of 50 to 200 μM.^152^ For the apomorphine sensor, four resin-based hollow MNs filled with unmodified carbon paste, rhodium nanoparticle containing carbon paste, or a silver wire were used to form a nonenzymatic sensor capable of detecting this medication. The sensor detected apomorphine *in vitro* with a limit of detection of 0.6 μM (using square-wave voltammetry) or 0.75 μM (using chronoamperometry). Moreover, the device showed good stability and antibiofouling properties against artificial ISF.^127^*Rawson et al*. engineered a sensor to detect the concentration of phenoxymethylpenicillin (PK), a common antibiotic, through the modification of metalized polycarbonate MN arrays with β-lactamase. The sensor was tested on 10 healthy human volunteers and showed to be capable of detecting phenoxymethylpenicillin with a limit of detection of 0·17 mg/L. Furthermore, the pharmacokinetic results obtained using the MN sensor were comparable to the ones obtained using microdialysis.^126^ Lastly, *Drăgan et al*. produced polyether ether ketone hollow MNs filled with silver or graphite paste and then modified with single-walled carbon nanotubes to detect 3,4-methylenedioxymethamphetamine (MDMA), an illicit drug. The sensor detected MDMA in artificial ISF within a linear range of 1 to 50 μM and a limit of detection of 0.75 μM.^129^ Overall, these devices showed excellent analytical performance, with high sensitivity and selectivity for their target analytes. Furthermore, the incorporation of the levodopa monitoring device^152^ with a portable wireless electroanalyzer demonstrated the ability of these medication monitoring devices to provide timely individualized feedback on the appropriate dosing regimen. In the future, we expect to see the extension of drug monitoring MN-based devices toward other analytes, both for rapid illicit drug screenings and for continuous monitoring of long-term dose-sensitive medications.

In conclusion, recently published MN-based biosensing devices have shown potential for the sensitive and selective analysis of a myriad of analytes *in situ*, from small biomolecules like alcohol to complex synthetic drugs like MDMA. The incorporation of some of these devices with wireless data transmission platforms further demonstrated their real-life applicability, helping users to make informed, data-based decisions about their health.

## Preclinical Testing

MNs have been tested in different *in vitro*, *ex vivo*, and *in vivo* animal models. Many authors use *ex vivo* models for insertion tests and to assess skin irritation,^134,135^ but excised animal skin has also been used, for instance, to test the ability of MNs to collect ISF. For instance, *Zheng et al*. tested the capacity of their osmolyte-composited swellable MNs to draw ISF *ex vivo* (in pig skin) and showed they were able to collect approximately 8 μL of ISF. The authors also tested these MNs *in vivo* (in mice), which collected a sufficient volume (∼4 μL) for the straightforward analysis of glucose via a MN-integrated electronic sensor.^18^ In another work, a MN-based diagnostic device built by adhering a MN patch to an electrochemical strip was tested *in vitro* (in a hydrogel model), and the results were comparable to blood glucose ranges for normal and diabetic status. However, the experiments *in vivo* resulted in a smaller electrochemical signal, which could still be correlated to blood glucose levels.^93^ MNs fabricated using a glucose-responsive polymer blend with conductive carbon nanotubes (sensing composite material) were also tested to draw ISF from rats (healthy and induced to have type 1 diabetes). The glucose concentrations measured with this device were comparable to blood samples analyzed using a glucometer, even after the MNs were worn for half a day. However, this MN array still needs to be paired with a separate reader or integrated with an electronic circuit to enable wireless data transmission to simplify personalized glucose monitoring.^153^ Moreover, gold nanoparticle swellable colorimetric MNs for glucose detection were validated for ISF extraction in female SD diabetic and healthy rats for 20 min. Color changes of the collected MN patch were analyzed using a smartphone camera and ImageJ software, and the concentrations of glucose in ISF obtained were consistent with blood glucose levels.^133^ Lastly, male NU/J athymic nude mice were used to assess the efficacy of a wearable sensor patch incorporating hydrogel MNs to measure in real-time both glucose and lactate concentrations in ISF. The sensor patch provided comparable measurements of glucose and lactate levels, particularly of glucose.^135^ Given this information, we believe that these integrated sensing platforms, combining MNs and advanced electrochemical sensing circuits, may soon contribute to improving disease management and overall health monitoring.

Overall, the preclinical validation experiments have shown promising ISF collection and biomarker detection.

## Clinical Trials

**Figure 4:**
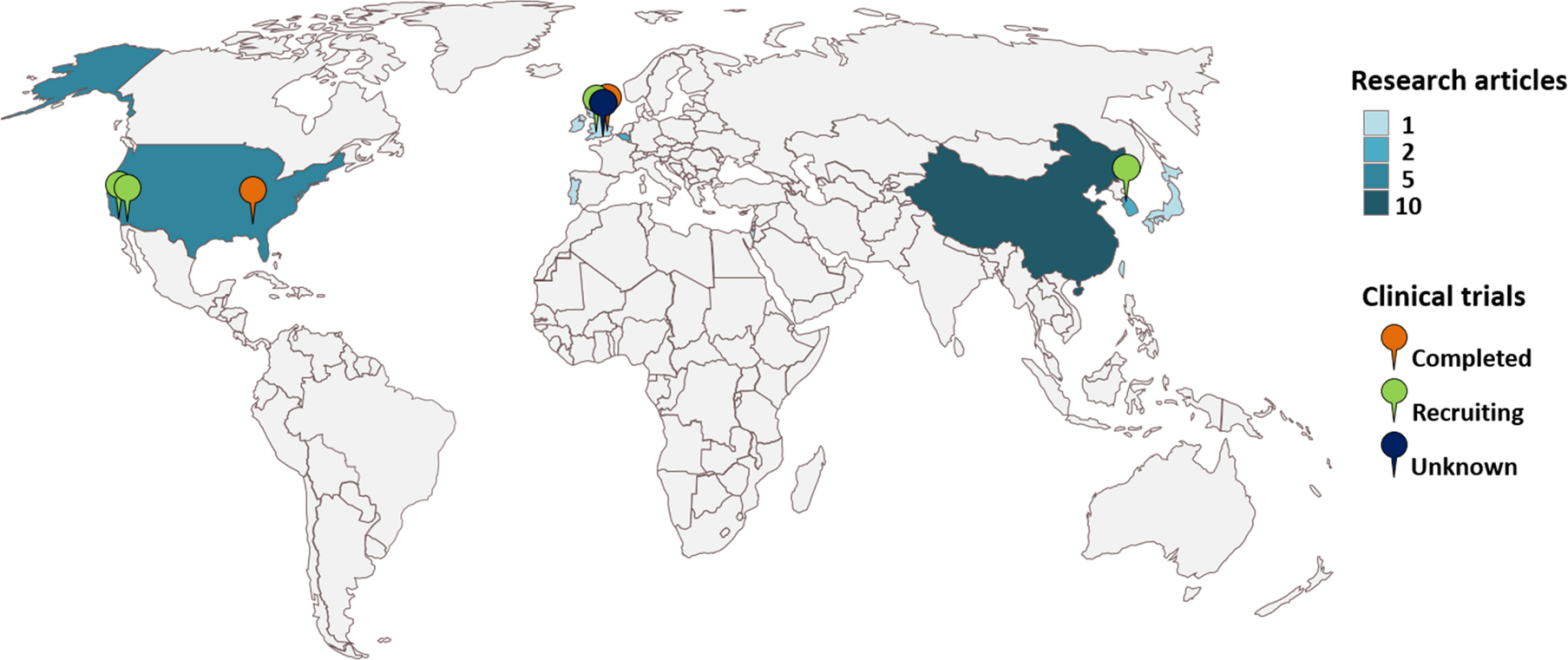
Geographical distribution of clinical trials involving microneedle-based health monitoring devices.

Ongoing and recruiting clinical trials will be briefly discussed (Figure 4). Taking into account the small number, all clinical trials, regardless of the material they are made of, are included in this section. To date, only two trials aiming for sample collection with MNs are listed at https://clinicaltrials.gov. There is one clinical trial recruiting participants with house dust mite allergic rhinitis (NCT05922176). Samples (RNA) will be collected with a minimally invasive technique, using a MN patch (no information is provided regarding the MN material used) as an alternative to skin biopsy and blood collection methods. This study will involve screening potential biomarkers to assess the response to immunotherapy, and to explore their utility as indicators for predicting the prognosis of immunotherapy in allergic diseases. The trial is recruiting participants aged 19 to 60 years, from all sexes, who have allergic rhinitis caused by the antigen of the American house dust mite, with moderate-severe persistent rhinitis, without any known skin diseases or allergic diseases. Samples will be collected from these participants before and after immunotherapy for transcriptomic profiling based on skin-extracted RNA samples for their comparison with normal skin samples.^154^ Then, there is a completed clinical trial related to glucose monitoring (NCT02682056) that enrolled 15 pediatric participants (7–18 years old) with a diagnosis of diabetes. Testing was performed using MN patches made from biocompatible polymers or metal, to collect ISF or an intravenous catheter and lancet to draw blood. The mean age of the participants was 16.8 (80% were white, 1 black or African American and 2 unknown). The study comprised glucose monitoring during 4 h, and other data was simultaneously collected (e.g., apprehension level regarding the sample collection varying between not afraid to very afraid, and pain level assessment). Monitored glucose levels were comparable between all the approaches used during the study testing period. The participants reported less apprehension with the MN patch than with the intravenous catheter, but still higher apprehension than when Lancet was used. No adverse effects were observed in any of the study participants.^155^

There is one clinical trial recruiting participants to use MNs (no information provided on the material of the hollow MNs that will be used) for levodopa monitoring (NCT04735627). In this trial, a levodopameter will be used in participants aged 48 to 85, which must meet the movement disorders society diagnostic criteria for clinically established Parkinson’s disease (mild, moderate, or severe), must be taking instant release oral carbidopa/levodopa therapy and also either not be taking or be on stable doses of other antiparkinsonian medications (e.g., dopamine agonists, monoamine oxidase B inhibitors or catecholamine O-methyl transferase inhibitors). The participants will receive oral or intravenous administered medication and then the MN device will measure the levodopa levels in ISF. These levels will be compared to plasma levodopa levels measured using high-performance liquid chromatography. The goal of this clinical trial is to allow patients with Parkinson’s disease to take proactive healthcare measures to maintain an optimal levodopa regimen.^156^

A clinical trial using a MN-based sensing device to monitor antibiotic concentrations was completed in 2020 (NCT03847610). This trial tested a polycarbonate MN array structure metallized to produce four independent electrodes^126^ for tracking phenoxymethylpenicillin concentrations in the plasma of 1o participants (mean age 42, 70% male) and compared it against the current gold standard (microdialysis and blood sampling). Although the accuracy of the device was not reported, none of the participants observed any adverse effects.^157^ Another clinical trial on closed-loop control of penicillin delivery is currently recruiting participants (NCT04053140). In this exploratory study, 20 healthy volunteers (>18 years old) will be enrolled and administered the drug. Three sets of tests will be conducted: 1) penicillin G_1200 mg administered every hour for collection of samples with a MN array, 2) penicillin G_2400 mg administered every 4 h for a MN array and closed-loop control delivery experiment, and 3) penicillin G_600 mg administered every hour for MN array and closed-loop control delivery studies. The polycarbonate MN biosensor metallized with chrome,^148^ will be sited peripherally. In study experiments 2 and 3, the MN arrays inserted will be collected after the duration of the study and used to titrate the benzylpenicillin dosage according to the pharmacokinetics and pharmacodynamics target. The main aim is to study the feasibility of the penicillin MN biosensor technology linked with a closed-loop automated delivery of antibiotics.^158^

A trial measuring methadone concentration on ISF using silicon microneedles^159^ is recruiting participants (aged 18–70) taking methadone for chronic pain (at least one dose of 10 mg per week, taken as prescribed in the last 4 days before consent to participate) (NCT05981573). This pilot study aims to assess if a physician can 1) recognize the peak of methadone dose use *ex vivo* in a remote medication monitor and 2) determine the status of prescribed dose taking over time. The ISF collected sample will be assayed electrochemically and the results compared to methadone concentrations in the blood detected using liquid chromatography, mass spectroscopy, and differential pulse voltammetry.^160^

There is also a clinical trial on lactate monitoring using MNs (no information on the MN material) in the clinicaltrials.org list (NCT04238611), however, its status is unknown, and the last update on the Web site was done in August 2021. This may be due to a delay in the recruiting of the participants. Therefore, although the status is presently unclear, we opted to include this trial in our review. The goal is to validate a MN-based device for the continuous measurement of lactate during exercise and compare its precision and accuracy with blood measurements. In addition, the acceptability in terms of pain, comfort, physical restriction, and skin sensation will be evaluated.^161^

The results of these trials will provide further knowledge on the future potential application of MN biosensing technologies in healthcare monitoring.

Clinical trials offer valuable insights into the potential applications of MN sensing technologies, and of their feasibility and efficacy in real-world clinical settings. Despite some trials being ongoing or having unknown statuses, their inclusion underscores the importance of exploring and validating MN-based technologies for continuous health monitoring.

## Microneedle Market

The global market for MNs is witnessing continuous growth. In 2023, the market estimates were expected to reach USD 768.9 million up to USD 2.81 billion, with the compound annual growth rate (CAGR) varying between 6.83% (2023–2028), and 6.6% (2023–2033).^162,163^ The drivers for this market revenue growth are related to the innovative delivery of medicinal formulations. The global market is segmented by type of product, application, and region. Based on type, the market is segmented into patch, rollers, radiofrequency, and laser MNs, and based on MN needle material, the market segments into metal, glass, polymer, and silicon.^164^ The solid MN segment dominates the market, witnessing an increase in the market of polymeric MNs due to the advancements in these systems as substitutes to conventional hypodermic injection for transdermal drug delivery.^165,166^ Based on application insights, the global market is segmented into skin rejuvenation, alopecia, sun damage, acne, scars, and spots. However, the increasing incidence of diabetes is driving the industry demand for minimally invasive and pain-free administration approaches. Moreover, technological advancements and the widespread adoption of smart devices, coupled with the integration of AI and data analytics, are also driving market growth.^165^ The main players in the MN market include companies such as Becton, Dickinson and Co., Cynosure, Candela Corp, and Vaxess Technologies Inc.^167^ In terms of market share, North America holds the highest share of this market. To date, there is no market estimate for MNs used in biosensing applications, but they are expected to be positioned in the same segment as polymeric MNs.

The global point-of-care diagnostics market was valued at USD 40.61 billion (2022) and is expected to expand at a CAGR of 6.5% from 2023 to 2030.^168^ The drivers for this growth are the rising prevalence of target diseases, increase in funding from multiple sources, rapid disease identification, and monitoring with reduced barriers to care. In this POC market, we find kits for glucose monitoring, infectious disease testing, and urine analysis, among others. Major market players in the POC market are intensifying their efforts to incorporate cutting-edge technologies like AI in the production of diagnostic tests, aiming to enhance efficiency (e.g., lab-on-a-chip platforms, wearable technology, and innovations in smartphone-based technology).^169^ MN-based sensing devices will soon have an impact on this market.

There are a few companies, predominantly in Asia, working with polymeric microneedles (Table 2). Most of these companies work with transdermal drug delivery products to replace the conventional injection approach or transdermal systems. These companies are pursuing their R&D with different medical-grade polymers (e.g., HA, polyglycolic acid, and poly(methyl methacrylate)) The materials being used by these companies account for nondegradable and biodegradable polymers. Although the companies identified in Table 2 mostly work on drug delivery, we identified a few companies using polymeric MNs for skin care [Raphas (Korea), CosMED Pharmaceuticals (Japan), and MITI Systems (Korea)]. There are some commercially available HA-based MN products on the market for skin care (e.g., Microcone, MicroHyala, and gMJETTM), but the vast majority of companies commercialize products for drug delivery (e.g., MIMIX patch and MPatch), or fabricate tailored services for their customers (e.g., the company Innoture, England). In terms of IP, most companies have the MN composition, fabrication, and application(s) protected. There are other companies also in this space that work with alternative MN materials (e.g., stainless steel, titanium, and ceramics). These nonpolymeric MN devices are used for varied applications including skin care, drug delivery, blood collection devices, and metabolite monitoring. According to our research, we did not find any commercially available MN device focused on monitoring and diagnostics. However, we anticipate that a few products will become closer to the market once the clinical trials have finished.

**Table 2:** Current List of Companies Commercializing Products Integrating Polymeric Microneedles

Company	Country	Products	Microneedle material
Raphas^170^	Korea	Microcone (8 Acropass beauty patches and 1 Therapass medical device)	Hyaluronic acid
MITI Systems^171^		Eye patch, spot patch, and sMTS EGF Oil Scrub Essence	
CosMED Pharmaceuticals^172^	Japan	MicroHyala and gMJETTM	
Think-Lands^173^		Information not available	Polyglycolic acid
Micropoint Technologies^174^	Singapore	Micropoint Patch and MPatch	Polyurethane, polypropylene, polyethylene, polystyrene, poly(methyl methacrylate), polycarbonate, hyaluronic acid, polyvinylpyrrolidone, carboxymethyl cellulose, . . .
Vaxess^175^	USA	MIMIX patch	Silk fibroin proteins
LTS^176^	Germany	Microneedle Patch	Polymers, including polyvinylpyrrolidone and polyvinyl alcohol
Innoture^177^	England	Customizable coated, solid, or dissolvable microneedles	Polymers (more specific information not available)

## Future Trends and Challenges

Throughout this review, we have shown that there is a great deal of interest in polymeric MNs for health monitoring. Considerable work has been published demonstrating the safety and efficacy of polymeric MNs for ISF collection and analysis, and there are already two completed trials and several others ongoing using MNs for diagnostics. Moreover, there are eight companies fabricating products based on polymeric MNs and two companies commercializing MN-based devices for biosensing purposes. As such, and given that both the MNs and POC devices industries represent multibillion-dollar market sectors expected to sustain a growing trajectory in the next decade, we expect the introduction of MN-based devices in the market in the next few years. Furthermore, unlike MNs for drug delivery, MNs for diagnostics do not need to follow a combined product approval path to obtain regulatory approval. Instead, they can be marked as medical devices,^178^ facilitating the approval process and reducing the time required to place these devices in the market. Future research in polymeric MN monitoring must continue to focus on advancing multiplexed analyte sensing systems to provide comprehensive health and disease monitoring, enhance diagnostic capabilities, and enable personalized healthcare. Additional effort is also expected on the integration of sensing components into polymeric MNs to enable more compact and portable devices for diagnostics. There is also a drive to develop wearable and patch-based systems for extended use. We anticipate that most, if not all, future polymeric MN-based sensing devices will incorporate wireless communication and connectivity features, allowing for continuous data transmission to smartphones or cloud-based platforms, and enabling remote real-time data monitoring and feedback, which will empower users to make informed decisions about their health. Also, integration with artificial intelligence and machine learning algorithms is foreseen for advanced data analysis, and interpretation (e.g., pattern identification and trend prediction) for personalized data-based health recommendations. Research efforts are also expected to advance long-term monitoring, potentially incorporating implantable MNs for continuous biomarker surveillance, offering significant advantages for managing chronic diseases. Anticipated developments include a transition toward biocompatible, biodegradable materials, alongside progress in nanomaterials and functional polymers. Integration of smart materials and responsive sensors into MNs is envisioned to enable dynamic adjustments in response to physiological changes, thus enhancing monitoring accuracy and reliability. Moreover, it is imperative to continue enhancing sensing performance through design, surface chemistry, and sensor integration for improved sensitivity, selectivity, and response time.

Despite promising, polymeric MN-based monitoring is challenged by stability, durability, and reliability of the integrated sensors due to the mechanical stresses encountered during wear, including long-term wear. Furthermore, their long-term adherence to skin might change, which may cause discomfort, and even pain, if the patch becomes loose. Biofouling problems also need to be carefully addressed at the concept stage to minimize these events. Furthermore, there are several obstacles to the translation of academic MN monitoring research into marketable products. For instance, scaling up of the manufacturing process to produce MNs with consistent quality in large quantities. This will require investing in cost-effective scalable techniques, such as roll-to-roll manufacturing. This can be challenging, because the materials used in scalable processes, might not be the same ones used at the research level, which will demand search for alternative biocompatible materials, and several iterations to optimize the scalable manufactured devices. In addition, regulatory agencies, such as the European Medicines Agency (EMA) and the United States Food and Drug Administration (FDA), may require adherence to Good Manufacturing Practices (GMP) guidelines to ensure that the devices are consistently produced and controlled according to quality standards. This will implicate considerable additional costs related, for example, to the establishment of GMP-compliant facilities and to an increase in operational costs. Besides, GMP compliance entails training of personnel on GMP principles throughout the organization, and thorough and complex documentation recording (e.g., standard operating procedures, batch records, quality control tests) which is resource-intensive. However, even if GMP compliance is not mandated, implementing quality management practices and adhering to relevant standards can help ensure the safety, reliability, and effectiveness of the devices. Finally, regulatory approval of the MN monitoring devices produced might also be a hurdle because this is a lengthy and complex process, which requires rigorous preclinical and clinical testing.

In conclusion, polymeric MN-based monitoring devices hold the promise of revolutionizing health diagnostics, providing rapid and minimally invasive testing beyond traditional laboratory confines. In addition, further advancements in fabrication techniques, sensing capabilities, and clinical validation are expected to drive the translation of MN technology into practical clinical applications. This advancement has the potential to significantly enhance healthcare accessibility, particularly in remote or resource-limited regions.

## Vocabulary

AA: acrylic acid
CAGR: compound annual growth rate
CEA: carcinoembryonic antigen
CKD: chronic kidney disease
EBV: Epstein–Barr virus
EMA: European Medicines Agency
FDA: United States Food and Drug Administration
GelMA: gelatin methacrylate
GMP: Good Manufacturing Practices
GOx: glucose oxidase
HA: hyaluronic acid
ISF: interstitial fluid
MeHA: methacrylated hyaluronic acid
MN: microneedle
PDMS: polydimethylsiloxane
SERS: surface-enhanced Raman scattering

## References

[ref1] Chambers R. Microdissection Studies, Iii. Some Problems in the Maturation and Fertilization of the Echinoderm Egg. Biol. Bull. 1921, 41 (6), 318–350. 10.2307/1536756.

[ref2] Gerstel M. S.; Place V. A. Drug Delivery Device. US3964482A, June 22, 1976. https://patents.google.com/patent/US3964482A/en (accessed 2024–01–02).

[ref3] Pistor M. L. P. Device for Cutaneous Therapeutic Treatment. US3918449A, November 11, 1975. https://patents.google.com/patent/US3918449A/en (accessed 2024–01–02).

[ref4] Henry S.; McAllister D. V.; Allen M. G.; Prausnitz M. R. Microfabricated Microneedles: A Novel Approach to Transdermal Drug Delivery. J. Pharm. Sci. 1998, 87 (8), 922–925. 10.1021/js980042+. 9687334

[ref5] McAllister D. V.; Wang P. M.; Davis S. P.; Park J.-H.; Canatella P. J.; Allen M. G.; Prausnitz M. R. Microfabricated Needles for Transdermal Delivery of Macromolecules and Nanoparticles: Fabrication Methods and Transport Studies. Proc. Natl. Acad. Sci. U. S. A. 2003, 100 (24), 13755–13760. 10.1073/pnas.2331316100. 14623977 PMC283494

[ref6] Mikszta J. A.; Alarcon J. B.; Brittingham J. M.; Sutter D. E.; Pettis R. J.; Harvey N. G. Improved Genetic Immunization via Micromechanical Disruption of Skin-Barrier Function and Targeted Epidermal Delivery. Nat. Med. 2002, 8 (4), 415–419. 10.1038/nm0402-415. 11927950

[ref7] Friedel M.; Thompson I. A. P.; Kasting G.; Polsky R.; Cunningham D.; Soh H. T.; Heikenfeld J. Opportunities and Challenges in the Diagnostic Utility of Dermal Interstitial Fluid. Nat. Biomed. Eng. 2023, 7 (12), 1541–1555. 10.1038/s41551-022-00998-9. 36658344

[ref8] Sempionatto J. R.; Lasalde-Ramírez J. A.; Mahato K.; Wang J.; Gao W. Wearable Chemical Sensors for Biomarker Discovery in the Omics Era. Nat. Rev. Chem. 2022, 6 (12), 899–915. 10.1038/s41570-022-00439-w. 37117704 PMC9666953

[ref9] Makvandi P.; Kirkby M.; Hutton A. R. J.; Shabani M.; Yiu C. K. Y.; Baghbantaraghdari Z.; Jamaledin R.; Carlotti M.; Mazzolai B.; Mattoli V.; Donnelly R. F. Engineering Microneedle Patches for Improved Penetration: Analysis, Skin Models and Factors Affecting Needle Insertion. Nano-Micro Lett. 2021, 13 (1), 93 10.1007/s40820-021-00611-9. PMC8006208 34138349

[ref10] Pires L. R.; Vinayakumar K. B.; Turos M.; Miguel V.; Gaspar J. A Perspective on Microneedle-Based Drug Delivery and Diagnostics in Paediatrics. J. Pers. Med. 2019, 9 (4), 49 10.3390/jpm9040049. 31731656 PMC6963643

[ref11] Lutton R. E. M.; Moore J.; Larrañeta E.; Ligett S.; Woolfson A. D.; Donnelly R. F. Microneedle Characterisation: The Need for Universal Acceptance Criteria and GMP Specifications When Moving towards Commercialisation. Drug Delivery Transl. Res. 2015, 5 (4), 313–331. 10.1007/s13346-015-0237-z. 26022578

[ref12] Wesley N. O.; Maibach H. I. Racial (Ethnic) Differences in Skin Properties. Am. J. Clin. Dermatol. 2003, 4 (12), 843–860. 10.2165/00128071-200304120-00004. 14640777

[ref13] Vesper H. W.; Wang P. M.; Archibold E.; Prausnitz M. R.; Myers G. L. Assessment of Trueness of a Glucose Monitor Using Interstitial Fluid and Whole Blood as Specimen Matrix. Diabetes Technol. Ther. 2006, 8 (1), 76–80. 10.1089/dia.2006.8.76. 16472053

[ref14] Wang P. M.; Cornwell M.; Prausnitz M. R. Minimally Invasive Extraction of Dermal Interstitial Fluid for Glucose Monitoring Using Microneedles. Diabetes Technol. Ther. 2005, 7 (1), 131–141. 10.1089/dia.2005.7.131. 15738711

[ref15] Kolluru C.; Williams M.; Chae J.; Prausnitz M. R. Recruitment and Collection of Dermal Interstitial Fluid Using a Microneedle Patch. Adv. Healthc. Mater. 2019, 8 (3), 1801262 10.1002/adhm.201801262. PMC6394862 30609270

[ref16] Laszlo E.; De Crescenzo G.; Nieto-Argüello A.; Banquy X.; Brambilla D. Superswelling Microneedle Arrays for Dermal Interstitial Fluid (Prote)Omics. Adv. Funct. Mater. 2021, 31 (46), 2106061 10.1002/adfm.202106061.

[ref17] Mukerjee E. V.; Collins S. D.; Isseroff R. R.; Smith R. L. Microneedle Array for Transdermal Biological Fluid Extraction and in Situ Analysis. Sens. Actuators Phys. 2004, 114 (2), 267–275. 10.1016/j.sna.2003.11.008.

[ref18] Zheng M.; Wang Z.; Chang H.; Wang L.; Chew S. W. T.; Lio D. C. S.; Cui M.; Liu L.; Tee B. C. K.; Xu C. Osmosis-Powered Hydrogel Microneedles for Microliters of Skin Interstitial Fluid Extraction within Minutes. Adv. Healthc. Mater. 2020, 9 (10), 1901683 10.1002/adhm.201901683. 32351042

[ref19] Chen J.; Wang M.; Ye Y.; Yang Z.; Ruan Z.; Jin N. Fabrication of Sponge-Forming Microneedle Patch for Rapidly Sampling Interstitial Fluid for Analysis. Biomed. Microdevices 2019, 21 (3), 63 10.1007/s10544-019-0413-x. 31273475

[ref20] Strambini L. M.; Longo A.; Scarano S.; Prescimone T.; Palchetti I.; Minunni M.; Giannessi D.; Barillaro G. Self-Powered Microneedle-Based Biosensors for Pain-Free High-Accuracy Measurement of Glycaemia in Interstitial Fluid. Biosens. Bioelectron. 2015, 66, 162–168. 10.1016/j.bios.2014.11.010. 25601169

[ref21] Ma S.; Li J.; Pei L.; Feng N.; Zhang Y. Microneedle-Based Interstitial Fluid Extraction for Drug Analysis: Advances, Challenges, and Prospects. J. Pharm. Anal. 2023, 13 (2), 111–126. 10.1016/j.jpha.2022.12.004. 36908860 PMC9999301

[ref22] El-Laboudi A.; Oliver N. S.; Cass A.; Johnston D. Use of Microneedle Array Devices for Continuous Glucose Monitoring: A Review. Diabetes Technol. Ther. 2013, 15 (1), 101–115. 10.1089/dia.2012.0188. 23234256

[ref23] Liu Y.; Yu Q.; Luo X.; Yang L.; Cui Y. Continuous Monitoring of Diabetes with an Integrated Microneedle Biosensing Device through 3D Printing. Microsyst. Nanoeng. 2021, 7 (1), 1–12. 10.1038/s41378-021-00302-w. 34631143 PMC8481261

[ref24] Corrie S. R.; Fernando G. J. P.; Crichton M. L.; Brunck M. E. G.; Anderson C. D.; Kendall M. A. F. Surface-Modified Microprojection Arrays for Intradermal Biomarker Capture, with Low Non-Specific Protein Binding. Lab. Chip 2010, 10 (20), 2655–2658. 10.1039/c0lc00068j. 20820632

[ref25] Teymourian H.; Moonla C.; Tehrani F.; Vargas E.; Aghavali R.; Barfidokht A.; Tangkuaram T.; Mercier P. P.; Dassau E.; Wang J. Microneedle-Based Detection of Ketone Bodies along with Glucose and Lactate: Toward Real-Time Continuous Interstitial Fluid Monitoring of Diabetic Ketosis and Ketoacidosis. Anal. Chem. 2020, 92 (2), 2291–2300. 10.1021/acs.analchem.9b05109. 31874029

[ref26] He R.; Liu H.; Fang T.; Niu Y.; Zhang H.; Han F.; Gao B.; Li F.; Xu F. A Colorimetric Dermal Tattoo Biosensor Fabricated by Microneedle Patch for Multiplexed Detection of Health-Related Biomarkers. Adv. Sci. 2021, 8 (24), 2103030 10.1002/advs.202103030. PMC8693053 34719884

[ref27] Saifullah K. M.; Faraji Rad Z. Sampling Dermal Interstitial Fluid Using Microneedles: A Review of Recent Developments in Sampling Methods and Microneedle-Based Biosensors. Adv. Mater. Interfaces 2023, 10 (10), 2201763 10.1002/admi.202201763.

[ref28] Bollella P.; Sharma S.; Cass A. E. G.; Antiochia R. Microneedle-Based Biosensor for Minimally-Invasive Lactate Detection. Biosens. Bioelectron. 2019, 123, 152–159. 10.1016/j.bios.2018.08.010. 30177422

[ref29] Hu Y.; Chatzilakou E.; Pan Z.; Traverso G.; Yetisen A. K. Microneedle Sensors for Point-of-Care Diagnostics. Adv. Sci. 2024, 11, 2306560 10.1002/advs.202306560. PMC10966570 38225744

[ref30] Poudineh M. Microneedle Assays for Continuous Health Monitoring: Challenges and Solutions. ACS Sens. 2024, 9 (2), 535–542. 10.1021/acssensors.3c02279. 38350235

[ref31] Chang H.; Zheng M.; Yu X.; Than A.; Seeni R. Z.; Kang R.; Tian J.; Khanh D. P.; Liu L.; Chen P.; Xu C. A Swellable Microneedle Patch to Rapidly Extract Skin Interstitial Fluid for Timely Metabolic Analysis. Adv. Mater. 2017, 29 (37), 1702243 10.1002/adma.201702243. 28714117

[ref32] Dervisevic M.; Dervisevic E.; Esser L.; Easton C. D.; Cadarso V. J.; Voelcker N. H. Wearable Microneedle Array-Based Sensor for Transdermal Monitoring of pH Levels in Interstitial Fluid. Biosens. Bioelectron. 2023, 222, 114955 10.1016/j.bios.2022.114955. 36462430

[ref33] Kim Y.-C.; Park J.-H.; Prausnitz M. R. Microneedles for Drug and Vaccine Delivery. Adv. Drug Delivery Rev. 2012, 64 (14), 1547–1568. 10.1016/j.addr.2012.04.005. PMC3419303 22575858

[ref34] Indermun S.; Luttge R.; Choonara Y. E.; Kumar P.; du Toit L. C.; Modi G.; Pillay V. Current Advances in the Fabrication of Microneedles for Transdermal Delivery. J. Controlled Release 2014, 185, 130–138. 10.1016/j.jconrel.2014.04.052. 24806483

[ref35] McCrudden M. T. C.; McAlister E.; Courtenay A. J.; González-Vázquez P.; Raj Singh T. R.; Donnelly R. F. Microneedle Applications in Improving Skin Appearance. Exp. Dermatol. 2015, 24 (8), 561–566. 10.1111/exd.12723. 25865925

[ref36] Ma G.; Wu C. Microneedle, Bio-Microneedle and Bio-Inspired Microneedle: A Review. J. Controlled Release 2017, 251, 11–23. 10.1016/j.jconrel.2017.02.011. 28215667

[ref37] Bhatnagar S.; Dave K.; Venuganti V. V. K. Microneedles in the Clinic. J. Controlled Release 2017, 260, 164–182. 10.1016/j.jconrel.2017.05.029. 28549948

[ref38] Ingrole R. S. J.; Gill H. S. Microneedle Coating Methods: A Review with a Perspective. J. Pharmacol. Exp. Ther. 2019, 370 (3), 555–569. 10.1124/jpet.119.258707. 31175217 PMC6806358

[ref39] Lee K. J.; Jeong S. S.; Roh D. H.; Kim D. Y.; Choi H.-K.; Lee E. H. A Practical Guide to the Development of Microneedle Systems - In Clinical Trials or on the Market. Int. J. Pharm. 2020, 573, 118778 10.1016/j.ijpharm.2019.118778. 31678394

[ref40] Nagarkar R.; Singh M.; Nguyen H. X.; Jonnalagadda S. A Review of Recent Advances in Microneedle Technology for Transdermal Drug Delivery. J. Drug Delivery Sci. Technol. 2020, 59, 101923 10.1016/j.jddst.2020.101923.

[ref41] Teymourian H.; Parrilla M.; Sempionatto J. R.; Montiel N. F.; Barfidokht A.; Van Echelpoel R.; De Wael K.; Wang J. Wearable Electrochemical Sensors for the Monitoring and Screening of Drugs. ACS Sens. 2020, 5 (9), 2679–2700. 10.1021/acssensors.0c01318. 32822166

[ref42] Sun H.; Zheng Y.; Shi G.; Haick H.; Zhang M. Wearable Clinic: From Microneedle-Based Sensors to Next-Generation Healthcare Platforms. Small 2023, 19 (51), 2207539 10.1002/smll.202207539. 36950771

[ref43] Teymourian H.; Tehrani F.; Mahato K.; Wang J. Lab under the Skin: Microneedle Based Wearable Devices. Adv. Healthc. Mater. 2021, 10 (17), 2002255 10.1002/adhm.202002255. 33646612

[ref44] Zheng M.; Sheng T.; Yu J.; Gu Z.; Xu C. Microneedle Biomedical Devices. Nat. Rev. Bioeng. 2024, 2, 324 10.1038/s44222-023-00141-6.

[ref45] Vora L. K.; Sabri A. H.; McKenna P. E.; Himawan A.; Hutton A. R. J.; Detamornrat U.; Paredes A. J.; Larrañeta E.; Donnelly R. F. Microneedle-Based Biosensing. Nat. Rev. Bioeng. 2024, 2 (1), 64–81. 10.1038/s44222-023-00108-7.

[ref46] Dervisevic M.; Alba M.; Prieto-Simon B.; Voelcker N. H. Skin in the Diagnostics Game: Wearable Biosensor Nano- and Microsystems for Medical Diagnostics. Nano Today 2020, 30, 100828 10.1016/j.nantod.2019.100828.

[ref47] Hu Y.; Chatzilakou E.; Pan Z.; Traverso G.; Yetisen A. K. Microneedle Sensors for Point-of-Care Diagnostics. Adv. Sci. 2024, 11, 2306560 10.1002/advs.202306560. PMC10966570 38225744

[ref48] García-Guzmán J. J.; Pérez-Ràfols C.; Cuartero M.; Crespo G. A. Microneedle Based Electrochemical (Bio)Sensing: Towards Decentralized and Continuous Health Status Monitoring. TrAC Trends Anal. Chem. 2021, 135, 116148 10.1016/j.trac.2020.116148.

[ref49] Starlin Chellathurai M.; Mahmood S.; Mohamed Sofian Z.; Wan Hee C.; Sundarapandian R.; Ahamed H. N.; Kandasamy C. S.; Hilles A. R.; Hashim N. M.; Janakiraman A. K. Biodegradable Polymeric Insulin Microneedles - a Design and Materials Perspective Review. Drug Delivery 2024, 31 (1), 2296350 10.1080/10717544.2023.2296350. 38147499 PMC10763835

[ref50] Mulkutkar M.; Damani M.; Sawarkar S. Polymeric Microneedles for the Eye: An Overview of Advances and Ocular Applications for Minimally Invasive Drug Delivery. Eur. J. Pharm. Biopharm. 2024, 197, 114209 10.1016/j.ejpb.2024.114209. 38336234

[ref51] Chen B. Z.; He Y. T.; Zhao Z. Q.; Feng Y. H.; Liang L.; Peng J.; Yang C. Y.; Uyama H.; Shahbazi M.-A.; Guo X. D. Strategies to Develop Polymeric Microneedles for Controlled Drug Release. Adv. Drug Delivery Rev. 2023, 203, 115109 10.1016/j.addr.2023.115109. 39492421

[ref52] Khalid R.; Mahmood S.; Mohamed Sofian Z.; Hilles A. R.; Hashim N. M.; Ge Y. Microneedles and Their Application in Transdermal Delivery of Antihypertensive Drugs—A Review. Pharmaceutics 2023, 15 (8), 2029 10.3390/pharmaceutics15082029. 37631243 PMC10459756

[ref53] Malek-Khatabi A.; Razavi M. S.; Abdollahi A.; Rahimzadeghan M.; Moammeri F.; Sheikhi M.; Tavakoli M.; Rad-Malekshahi M.; Rad Z. F. Recent Progress in PLGA-Based Microneedle-Mediated Transdermal Drug and Vaccine Delivery. Biomater. Sci. 2023, 11 (16), 5390–5409. 10.1039/D3BM00795B. 37387317

[ref54] Qi Z.; Yan Z.; Tan G.; Jia T.; Geng Y.; Shao H.; Kundu S. C.; Lu S. Silk Fibroin Microneedles for Transdermal Drug Delivery: Where Do We Stand and How Far Can We Proceed?. Pharmaceutics 2023, 15 (2), 355 10.3390/pharmaceutics15020355. 36839676 PMC9964088

[ref55] Karim Z.; Karwa P.; Hiremath S. R. R. Polymeric Microneedles for Transdermal Drug Delivery- a Review of Recent Studies. J. Drug Delivery Sci. Technol. 2022, 77, 103760 10.1016/j.jddst.2022.103760.

[ref56] Mbituyimana B.; Ma G.; Shi Z.; Yang G. Polymeric Microneedles for Enhanced Drug Delivery in Cancer Therapy. Biomater. Adv. 2022, 142, 213151 10.1016/j.bioadv.2022.213151. 36244246

[ref57] Vora L. K.; Moffatt K.; Tekko I. A.; Paredes A. J.; Volpe-Zanutto F.; Mishra D.; Peng K.; Raj Singh Thakur R.; Donnelly R. F. Microneedle Array Systems for Long-Acting Drug Delivery. Eur. J. Pharm. Biopharm. 2021, 159, 44–76. 10.1016/j.ejpb.2020.12.006. 33359666

[ref58] Kulkarni D.; Gadade D.; Chapaitkar N.; Shelke S.; Pekamwar S.; Aher R.; Ahire A.; Avhale M.; Badgule R.; Bansode R.; Bobade B. Polymeric Microneedles: An Emerging Paradigm for Advanced Biomedical Applications. Sci. Pharm. 2023, 91 (2), 27 10.3390/scipharm91020027.

[ref59] Luo X.; Yang L.; Cui Y. Microneedles: Materials, Fabrication, and Biomedical Applications. Biomed. Microdevices 2023, 25 (3), 20 10.1007/s10544-023-00658-y. 37278852 PMC10242236

[ref60] Cárcamo-Martínez A.; Mallon B.; Domínguez-Robles J.; Vora L. K.; Anjani Q. K.; Donnelly R. F. Hollow Microneedles: A Perspective in Biomedical Applications. Int. J. Pharm. 2021, 599, 120455 10.1016/j.ijpharm.2021.120455. 33676993

[ref61] Sil D.; Bhowmik S.; Patel P.; Kurmi B. D. Promising Role of Microneedles in Therapeutic and Biomedical Applications. J. Drug Delivery Sci. Technol. 2024, 91, 105273 10.1016/j.jddst.2023.105273.

[ref62] Kim S.; Lee M. S.; Yang H. S.; Jung J. H. Enhanced Extraction of Skin Interstitial Fluid Using a 3D Printed Device Enabling Tilted Microneedle Penetration. Sci. Rep. 2021, 11 (1), 14018 10.1038/s41598-021-93235-3. 34234204 PMC8263571

[ref63] Samant P. P.; Niedzwiecki M. M.; Raviele N.; Tran V.; Mena-Lapaix J.; Walker D. I.; Felner E. I.; Jones D. P.; Miller G. W.; Prausnitz M. R. Sampling Interstitial Fluid from Human Skin Using a Microneedle Patch. Sci. Transl. Med. 2020, 12 (571), eaaw0285 10.1126/scitranslmed.aaw0285. 33239384 PMC7871333

[ref64] Azizi Machekposhti S.; Nguyen A. K.; Vanderwal L.; Stafslien S.; Narayan R. J. Micromolding of Amphotericin-B-Loaded Methoxyethylene-Maleic Anhydride Copolymer Microneedles. Pharmaceutics 2022, 14 (8), 1551 10.3390/pharmaceutics14081551. 35893806 PMC9331399

[ref65] Ebrahiminejad V.; Faraji Rad Z.; Prewett P. D.; Davies G. J. Fabrication and Testing of Polymer Microneedles for Transdermal Drug Delivery. Beilstein J. Nanotechnol. 2022, 13, 629–640. 10.3762/bjnano.13.55. 35874440 PMC9273988

[ref66] Li J.; Liu B.; Zhou Y.; Chen Z.; Jiang L.; Yuan W.; Liang L. Fabrication of a Ti Porous Microneedle Array by Metal Injection Molding for Transdermal Drug Delivery. PloS One 2017, 12 (2), e0172043 10.1371/journal.pone.0172043. 28187179 PMC5302820

[ref67] Wu L.; Park J.; Kamaki Y.; Kim B. Optimization of the Fused Deposition Modeling-Based Fabrication Process for Polylactic Acid Microneedles. Microsyst. Nanoeng. 2021, 7, 58 10.1038/s41378-021-00284-9. 34567770 PMC8433210

[ref68] Xiang Z.; Liu J.; Lee C. A Flexible Three-Dimensional Electrode Mesh: An Enabling Technology for Wireless Brain-Computer Interface Prostheses. Microsyst. Nanoeng. 2016, 2, 16012 10.1038/micronano.2016.12. 31057819 PMC6444742

[ref69] Roh H.; Yoon Y. J.; Park J. S.; Kang D.-H.; Kwak S. M.; Lee B. C.; Im M. Fabrication of High-Density Out-of-Plane Microneedle Arrays with Various Heights and Diverse Cross-Sectional Shapes. Nano-Micro Lett. 2022, 14 (1), 24 10.1007/s40820-021-00778-1. PMC8656445 34888758

[ref70] Kathuria H.; Kang K.; Cai J.; Kang L. Rapid Microneedle Fabrication by Heating and Photolithography. Int. J. Pharm. 2020, 575, 118992 10.1016/j.ijpharm.2019.118992. 31884060

[ref71] Trautmann A.; Roth G.-L.; Nujiqi B.; Walther T.; Hellmann R. Towards a Versatile Point-of-Care System Combining Femtosecond Laser Generated Microfluidic Channels and Direct Laser Written Microneedle Arrays. Microsyst. Nanoeng. 2019, 5, 6 10.1038/s41378-019-0046-5. 31057933 PMC6387975

[ref72] Watanabe H.; Cardoso L.; Lalwani A. K.; Kysar J. W. A Dual Wedge Microneedle for Sampling of Perilymph Solution via Round Window Membrane. Biomed. Microdevices 2016, 18 (2), 24 10.1007/s10544-016-0046-2. 26888440 PMC5574191

[ref73] Vinayakumar K. B.; Silva M. D.; Martins A.; Mundy S.; González-Losada P.; Sillankorva S. Levofloxacin-Loaded Microneedles Produced Using 3D-Printed Molds for Klebsiella Pneumoniae Biofilm Control. Adv. Ther. 2023, 6 (6), 2200320 10.1002/adtp.202200320.

[ref74] Miller P. R.; Taylor R. M.; Tran B. Q.; Boyd G.; Glaros T.; Chavez V. H.; Krishnakumar R.; Sinha A.; Poorey K.; Williams K. P.; Branda S. S.; Baca J. T.; Polsky R. Extraction and Biomolecular Analysis of Dermal Interstitial Fluid Collected with Hollow Microneedles. Commun. Biol. 2018, 1 (1), 1–11. 10.1038/s42003-018-0170-z. 30374463 PMC6197253

[ref75] Lee K.; Jung H. Drawing Lithography for Microneedles: A Review of Fundamentals and Biomedical Applications. Biomaterials 2012, 33 (30), 7309–7326. 10.1016/j.biomaterials.2012.06.065. 22831855

[ref76] Liu Y.; Yu Q.; Ye L.; Yang L.; Cui Y. A Wearable, Minimally-Invasive, Fully Electrochemically-Controlled Feedback Minisystem for Diabetes Management. Lab. Chip 2023, 23 (3), 421–436. 10.1039/D2LC00797E. 36597970

[ref77] Simas M. V.; Olaniyan P. O.; Hati S.; Davis G. A. Jr; Anspach G.; Goodpaster J. V.; Manicke N. E.; Sardar R. Superhydrophobic Surface Modification of Polymer Microneedles Enables Fabrication of Multimodal Surface-Enhanced Raman Spectroscopy and Mass Spectrometry Substrates for Synthetic Drug Detection in Blood Plasma. ACS Appl. Mater. Interfaces 2023, 15 (40), 46681–46696. 10.1021/acsami.3c10174. 37769194

[ref78] Sharifuzzaman M.; Shin Y. D.; Yoo J.; Reza M. S.; kim Y.-R.; Park J. Y. An Oxygen-Insensitive and Minimally Invasive Polymeric Microneedle Sensor for Continuous and Wide-Range Transdermal Glucose Monitoring. Talanta 2023, 263, 124747 10.1016/j.talanta.2023.124747. 37267884

[ref79] Saadat-Moghaddam D.; Kim J.-H. A Microneedle Functionalized with Polyethyleneimine and Nanotubes for Highly Sensitive, Label-Free Quantification of DNA. Sensors 2017, 17 (8), 1883 10.3390/s17081883. 28812987 PMC5579740

[ref80] Matadh A. V.; Jakka D.; Pragathi S. G.; Rangappa S.; Shivakumar H. N.; Maibach H.; Reena N. M.; Murthy S. N. Polymer-Coated Polymeric (PCP) Microneedles for Controlled Dermal Delivery of 5-Fluorouracil. AAPS PharmSciTech 2023, 24 (1), 9 10.1208/s12249-022-02471-x. 36450897

[ref81] Zhu J.; Zhou X.; Kim H.-J.; Qu M.; Jiang X.; Lee K.; Ren L.; Wu Q.; Wang C.; Zhu X.; Tebon P.; Zhang S.; Lee J.; Ashammakhi N.; Ahadian S.; Dokmeci M. R.; Gu Z.; Sun W.; Khademhosseini A. Gelatin Methacryloyl Microneedle Patches for Minimally Invasive Extraction of Skin Interstitial Fluid. Small 2020, 16 (16), 1905910 10.1002/smll.201905910. PMC7182487 32101371

[ref82] Ito Y.; Inagaki Y.; Kobuchi S.; Takada K.; Sakaeda T. Therapeutic Drug Monitoring of Vancomycin in Dermal Interstitial Fluid Using Dissolving Microneedles. Int. J. Med. Sci. 2016, 13 (4), 271–276. 10.7150/ijms.13601. 27076783 PMC4829539

[ref83] Qiao Y.; Du J.; Ge R.; Lu H.; Wu C.; Li J.; Yang S.; Zada S.; Dong H.; Zhang X. A Sample and Detection Microneedle Patch for Psoriasis MicroRNA Biomarker Analysis in Interstitial Fluid. Anal. Chem. 2022, 94 (14), 5538–5545. 10.1021/acs.analchem.1c04401. 35315641

[ref84] He R.; Niu Y.; Li Z.; Li A.; Yang H.; Xu F.; Li F. A Hydrogel Microneedle Patch for Point-of-Care Testing Based on Skin Interstitial Fluid. Adv. Healthc. Mater. 2020, 9 (4), 1901201 10.1002/adhm.201901201. 31957291

[ref85] Xu N.; Zhang M.; Xu W.; Ling G.; Yu J.; Zhang P. Swellable PVA/PVP Hydrogel Microneedle Patches for the Extraction of Interstitial Skin Fluid toward Minimally Invasive Monitoring of Blood Glucose Level. Analyst 2022, 147 (7), 1478–1491. 10.1039/D1AN02288A. 35285841

[ref86] Sillankorva S.; Pires L.; Pastrana L. M.; Bañobre-López M. Antibiofilm Efficacy of the Pseudomonas Aeruginosa Pbunavirus vB_PaeM-SMS29 Loaded onto Dissolving Polyvinyl Alcohol Microneedles. Viruses 2022, 14 (5), 964 10.3390/v14050964. 35632706 PMC9143888

[ref87] Zhou Z.; Xing M.; Zhang S.; Yang G.; Gao Y. Process Optimization of Ca2+ Cross-Linked Alginate-Based Swellable Microneedles for Enhanced Transdermal Permeability: More Applicable to Acidic Drugs. Int. J. Pharm. 2022, 618, 121669 10.1016/j.ijpharm.2022.121669. 35306152

[ref88] Dervisevic M.; Alba M.; Yan L.; Senel M.; Gengenbach T. R.; Prieto-Simon B.; Voelcker N. H. Transdermal Electrochemical Monitoring of Glucose via High-Density Silicon Microneedle Array Patch. Adv. Funct. Mater. 2022, 32 (3), 2009850 10.1002/adfm.202009850.

[ref89] Song S.; Na J.; Jang M.; Lee H.; Lee H.-S.; Lim Y.-B.; Choi H.; Chae Y. A CMOS VEGF Sensor for Cancer Diagnosis Using a Peptide Aptamer-Based Functionalized Microneedle. IEEE Trans. Biomed. Circuits Syst. 2019, 13 (6), 1288–1299. 10.1109/TBCAS.2019.2954846. 31751251

[ref90] Mishra R. K.; Goud K. Y.; Li Z.; Moonla C.; Mohamed M. A.; Tehrani F.; Teymourian H.; Wang J. Continuous Opioid Monitoring along with Nerve Agents on a Wearable Microneedle Sensor Array. J. Am. Chem. Soc. 2020, 142 (13), 5991–5995. 10.1021/jacs.0c01883. 32202103

[ref91] Zheng H.; GhavamiNejad A.; GhavamiNejad P.; Samarikhalaj M.; Giacca A.; Poudineh M. Hydrogel Microneedle-Assisted Assay Integrating Aptamer Probes and Fluorescence Detection for Reagentless Biomarker Quantification. ACS Sens. 2022, 7 (8), 2387–2399. 10.1021/acssensors.2c01033. 35866892

[ref92] Donnelly R. F.; Woolfson A. D. Patient Safety and beyond: What Should We Expect from Microneedle Arrays in the Transdermal Delivery Arena?. Ther. Delivery 2014, 5 (6), 653–662. 10.4155/tde.14.29. 25090279

[ref93] Zheng M.; Zhang Y.; Hu T.; Xu C. A Skin Patch Integrating Swellable Microneedles and Electrochemical Test Strips for Glucose and Alcohol Measurement in Skin Interstitial Fluid. Bioeng. Transl. Med. 2023, 8 (5), e10413 10.1002/btm2.10413. 37693058 PMC10487322

[ref94] Kaushik S.; Hord A. H.; Denson D. D.; McAllister D. V.; Smitra S.; Allen M. G.; Prausnitz M. R. Lack of Pain Associated with Microfabricated Microneedles. Anesth. Analg. 2001, 92 (2), 502 10.1213/00000539-200102000-00041. 11159258

[ref95] Haq M. I.; Smith E.; John D. N.; Kalavala M.; Edwards C.; Anstey A.; Morrissey A.; Birchall J. C. Clinical Administration of Microneedles: Skin Puncture, Pain and Sensation. Biomed. Microdevices 2009, 11 (1), 35–47. 10.1007/s10544-008-9208-1. 18663579

[ref96] Van Damme P.; Oosterhuis-Kafeja F.; Van der Wielen M.; Almagor Y.; Sharon O.; Levin Y. Safety and Efficacy of a Novel Microneedle Device for Dose Sparing Intradermal Influenza Vaccination in Healthy Adults. Vaccine 2009, 27 (3), 454–459. 10.1016/j.vaccine.2008.10.077. 19022318

[ref97] Frew P. M.; Paine M. B.; Rouphael N.; Schamel J.; Chung Y.; Mulligan M. J.; Prausnitz M. R. Acceptability of an Inactivated Influenza Vaccine Delivered by Microneedle Patch: Results from a Phase I Clinical Trial of Safety, Reactogenicity, and Immunogenicity. Vaccine 2020, 38 (45), 7175–7181. 10.1016/j.vaccine.2020.07.064. 32792250

[ref98] Bal S. M.; Caussin J.; Pavel S.; Bouwstra J. A. In Vivo Assessment of Safety of Microneedle Arrays in Human Skin. Eur. J. Pharm. Sci. 2008, 35 (3), 193–202. 10.1016/j.ejps.2008.06.016. 18657610

[ref99] Hoesly F. J.; Borovicka J.; Gordon J.; Nardone B.; Holbrook J. S.; Pace N.; Ibrahim O.; Bolotin D.; Warycha M.; Kwasny M.; West D.; Alam M. Safety of a Novel Microneedle Device Applied to Facial Skin: A Subject- and Rater-Blinded, Sham-Controlled, Randomized Trial. Arch. Dermatol. 2012, 148 (6), 711–717. 10.1001/archdermatol.2012.280. 22431712

[ref100] Rouphael N. G.; Paine M.; Mosley R.; Henry S.; McAllister D. V.; Kalluri H.; Pewin W.; Frew P. M.; Yu T.; Thornburg N. J.; Kabbani S.; Lai L.; Vassilieva E. V.; Skountzou I.; Compans R. W.; Mulligan M. J.; Prausnitz M. R.; Beck A.; Edupuganti S.; Heeke S.; Kelley C.; Nesheim W. The Safety, Immunogenicity, and Acceptability of Inactivated Influenza Vaccine Delivered by Microneedle Patch (TIV-MNP 2015): A Randomised, Partly Blinded, Placebo-Controlled, Phase 1 Trial. Lancet 2017, 390 (10095), 649–658. 10.1016/S0140-6736(17)30575-5. 28666680 PMC5578828

[ref101] Chege M.; McConville A.; Davis J. Microneedle Drug Delivery Systems: Appraising Opportunities for Improving Safety and Assessing Areas of Concern. J. Chem. Health Saf. 2017, 24 (2), 6–14. 10.1016/j.jchas.2016.04.008.

[ref102] Zhu D. D.; Zhang X. P.; Zhang B. L.; Hao Y. Y.; Guo X. D. Safety Assessment of Microneedle Technology for Transdermal Drug Delivery: A Review. Adv. Ther. 2020, 3 (8), 2000033 10.1002/adtp.202000033.

[ref103] Larrañeta E.; Lutton R. E. M.; Woolfson A. D.; Donnelly R. F. Microneedle Arrays as Transdermal and Intradermal Drug Delivery Systems: Materials Science, Manufacture and Commercial Development. Mater. Sci. Eng. R Rep. 2016, 104, 1–32. 10.1016/j.mser.2016.03.001.

[ref104] Faraji Rad Z.; Prewett P. D.; Davies G. J. An Overview of Microneedle Applications, Materials, and Fabrication Methods. Beilstein J. Nanotechnol. 2021, 12, 1034–1046. 10.3762/bjnano.12.77. 34621614 PMC8450954

[ref105] Bhattarai D. P.; Pokharel P.; Xiao D. Surface Functionalization of Polymers. In Reactive and Functional Polymers Vol. Four: Surface, Interface, Biodegradability, Compostability and Recycling; Gutiérrez T. J., Ed.; Springer International Publishing: Cham, 2020; pp 5–34. 10.1007/978-3-030-52052-6_2.

[ref106] Lyu S.; Dong Z.; Xu X.; Bei H.-P.; Yuen H.-Y.; James Cheung C.-W.; Wong M.-S.; He Y.; Zhao X. Going below and beyond the Surface: Microneedle Structure, Materials, Drugs, Fabrication, and Applications for Wound Healing and Tissue Regeneration. Bioact. Mater. 2023, 27, 303–326. 10.1016/j.bioactmat.2023.04.003. 37122902 PMC10140753

[ref107] Zhu J.; Zhou X.; Libanori A.; Sun W. Microneedle-Based Bioassays. Nanoscale Adv. 2020, 2 (10), 4295–4304. 10.1039/D0NA00543F. 36132929 PMC9419780

[ref108] Huang H.; Qu M.; Zhou Y.; Cao W.; Huang X.; Sun J.; Sun W.; Zhou X.; Xu M.; Jiang X. A Microneedle Patch for Breast Cancer Screening via Minimally Invasive Interstitial Fluid Sampling. Chem. Eng. J. 2023, 472, 145036 10.1016/j.cej.2023.145036.

[ref109] Wang Z.; Luan J.; Seth A.; Liu L.; You M.; Gupta P.; Rathi P.; Wang Y.; Cao S.; Jiang Q.; Zhang X.; Gupta R.; Zhou Q.; Morrissey J. J.; Scheller E. L.; Rudra J. S.; Singamaneni S. Microneedle Patch for the Ultrasensitive Quantification of Protein Biomarkers in Interstitial Fluid. Nat. Biomed. Eng. 2021, 5 (1), 64–76. 10.1038/s41551-020-00672-y. 33483710 PMC8020465

[ref110] Fonseca D. F. S.; Costa P. C.; Almeida I. F.; Dias-Pereira P.; Correia-Sá I.; Bastos V.; Oliveira H.; Vilela C.; Silvestre A. J. D.; Freire C. S. R. Swellable Gelatin Methacryloyl Microneedles for Extraction of Interstitial Skin Fluid toward Minimally Invasive Monitoring of Urea. Macromol. Biosci. 2020, 20 (10), 2000195 10.1002/mabi.202000195. 33405374

[ref111] Takeuchi K.; Takama N.; Kinoshita R.; Okitsu T.; Kim B. Flexible and Porous Microneedles of PDMS for Continuous Glucose Monitoring. Biomed. Microdevices 2020, 22 (4), 79 10.1007/s10544-020-00532-1. 33141313

[ref112] Hsieh Y.-C.; Lin C.-Y.; Lin H.-Y.; Kuo C.-T.; Yin S.-Y.; Hsu Y.-H.; Yeh H.-F.; Wang J.; Wan D. Controllable-Swelling Microneedle-Assisted Ultrasensitive Paper Sensing Platforms for Personal Health Monitoring. Adv. Healthc. Mater. 2023, 12 (24), 2300321 10.1002/adhm.202300321. 37037493

[ref113] Al Sulaiman D.; Chang J. Y. H.; Bennett N. R.; Topouzi H.; Higgins C. A.; Irvine D. J.; Ladame S. Hydrogel-Coated Microneedle Arrays for Minimally Invasive Sampling and Sensing of Specific Circulating Nucleic Acids from Skin Interstitial Fluid. ACS Nano 2019, 13 (8), 9620–9628. 10.1021/acsnano.9b04783. 31411871 PMC6746174

[ref114] Zhang X.; Chen G.; Bian F.; Cai L.; Zhao Y. Encoded Microneedle Arrays for Detection of Skin Interstitial Fluid Biomarkers. Adv. Mater. 2019, 31 (37), 1902825 10.1002/adma.201902825. 31271485

[ref115] Wu J.; Liu Y.; Peng L.; Liu Q.; Wang D.; Jing X.; Hu Y.; Lin J.; Fu H.; Ji X.; Liu J.; Lv H.; Peng B.; Zhang B.; Guo L.; Wang S. A Plasmonic Fluor-Lightened Microneedle Array Enables Ultrasensitive Multitarget Whole Blood Diagnosis of Anemia in A Paper Origami-Based Device. Small 2023, 19 (26), 2300464 10.1002/smll.202300464. 36950741

[ref116] Luo X.; Yu Q.; Liu Y.; Gai W.; Ye L.; Yang L.; Cui Y. Closed-Loop Diabetes Minipatch Based on a Biosensor and an Electroosmotic Pump on Hollow Biodegradable Microneedles. ACS Sens. 2022, 7 (5), 1347–1360. 10.1021/acssensors.1c02337. 35442623

[ref117] Mota F. A. R.; Passos M. L. C.; Santos J. L. M.; Saraiva M. L. M. F. S. Comparative Analysis of Electrochemical and Optical Sensors for Detection of Chronic Wounds Biomarkers: A Review. Biosens. Bioelectron. 2024, 251, 116095 10.1016/j.bios.2024.116095. 38382268

[ref118] Jancev M.; Vissers T. A. C. M.; Visseren F. L. J.; van Bon A. C.; Serné E. H.; DeVries J. H.; de Valk H. W.; van Sloten T. T. Continuous Glucose Monitoring in Adults with Type 2 Diabetes: A Systematic Review and Meta-Analysis. Diabetologia 2024, 67, 798 10.1007/s00125-024-06107-6. 38363342 PMC10954850

[ref119] Ehrhardt N.; Al Zaghal E. Behavior Modification in Prediabetes and Diabetes: Potential Use of Real-Time Continuous Glucose Monitoring. J. Diabetes Sci. Technol. 2019, 13 (2), 271–275. 10.1177/1932296818790994. 30066574 PMC6399786

[ref120] Rasmussen L.; Christensen M. L.; Poulsen C. W.; Rud C.; Christensen A. S.; Andersen J. R.; Kampmann U.; Ovesen P. G. Effect of High Versus Low Carbohydrate Intake in the Morning on Glycemic Variability and Glycemic Control Measured by Continuous Blood Glucose Monitoring in Women with Gestational Diabetes Mellitus-A Randomized Crossover Study. Nutrients 2020, 12 (2), 475 10.3390/nu12020475. 32069857 PMC7071236

[ref121] Okabayashi T.; Shima Y.; Sumiyoshi T.; Kozuki A.; Ito S.; Ogawa Y.; Kobayashi M.; Hanazaki K. Diagnosis and Management of Insulinoma. World J. Gastroenterol. WJG 2013, 19 (6), 829–837. 10.3748/wjg.v19.i6.829. 23430217 PMC3574879

[ref122] Son D.-H.; Lee H. S.; Lee Y.-J.; Lee J.-H.; Han J.-H. Comparison of Triglyceride-Glucose Index and HOMA-IR for Predicting Prevalence and Incidence of Metabolic Syndrome. Nutr. Metab. Cardiovasc. Dis. NMCD 2022, 32 (3), 596–604. 10.1016/j.numecd.2021.11.017. 35090800

[ref123] Salehidoost R.; Korbonits M. Glucose and Lipid Metabolism Abnormalities in Cushing’s Syndrome. J. Neuroendocrinol. 2022, 34 (8), e13143 10.1111/jne.13143. 35980242

[ref124] Parrilla M.; Detamornrat U.; Domínguez-Robles J.; Donnelly R. F.; De Wael K. Wearable Hollow Microneedle Sensing Patches for the Transdermal Electrochemical Monitoring of Glucose. Talanta 2022, 249, 123695 10.1016/j.talanta.2022.123695. 35728453

[ref125] Barrett C.; O’Sullivan F.; Barry S.; Grygoryev K.; O’Gorman D.; O’Mahony C.; O’Riordan A. Novel Surface Modified Polymer Microneedle Based Biosensors for Interstitial Fluid Glucose Detection. 2019 IEEE SENSORS 2019, 1–4. 10.1109/SENSORS43011.2019.8956509.

[ref126] Rawson T. M.; Gowers S. A. N.; Freeman D. M. E.; Wilson R. C.; Sharma S.; Gilchrist M.; MacGowan A.; Lovering A.; Bayliss M.; Kyriakides M.; Georgiou P.; Cass A. E. G.; O’Hare D.; Holmes A. H. Microneedle Biosensors for Real-Time, Minimally Invasive Drug Monitoring of Phenoxymethylpenicillin: A First-in-Human Evaluation in Healthy Volunteers. Lancet Digit. Health 2019, 1 (7), e335–e343. 10.1016/S2589-7500(19)30131-1. 33323208

[ref127] Goud K. Y.; Mahato K.; Teymourian H.; Longardner K.; Litvan I.; Wang J. Wearable Electrochemical Microneedle Sensing Platform for Real-Time Continuous Interstitial Fluid Monitoring of Apomorphine: Toward Parkinson Management. Sens. Actuators B Chem. 2022, 354, 131234 10.1016/j.snb.2021.131234.

[ref128] Sang M.; Cho M.; Lim S.; Min I. S.; Han Y.; Lee C.; Shin J.; Yoon K.; Yeo W.-H.; Lee T.; Won S. M.; Jung Y.; Heo Y. J.; Yu K. J. Fluorescent-Based Biodegradable Microneedle Sensor Array for Tether-Free Continuous Glucose Monitoring with Smartphone Application. Sci. Adv. 2023, 9 (22), eadh1765 10.1126/sciadv.adh1765. 37256939 PMC10413647

[ref129] Drăgan A.-M.; Parrilla M.; Cambré S.; Domínguez-Robles J.; Detamornrat U.; Donnelly R. F.; Oprean R.; Cristea C.; De Wael K. Microneedle Array-Based Electrochemical Sensor Functionalized with SWCNTs for the Highly Sensitive Monitoring of MDMA in Interstitial Fluid. Microchem. J. 2023, 193, 109257 10.1016/j.microc.2023.109257.

[ref130] Dervisevic M.; Voelcker N. H. Microneedles with Recessed Microcavities for Electrochemical Sensing in Dermal Interstitial Fluid. ACS Mater. Lett. 2023, 5 (7), 1851–1858. 10.1021/acsmaterialslett.3c00441.

[ref131] Zhao L.; Wen Z.; Jiang F.; Zheng Z.; Lu S. Silk/Polyols/GOD Microneedle Based Electrochemical Biosensor for Continuous Glucose Monitoring. RSC Adv. 2020, 10 (11), 6163–6171. 10.1039/C9RA10374K. 35496012 PMC9049677

[ref132] Zeng Y.; Wang J.; Wang Z.; Chen G.; Yu J.; Li S.; Li Q.; Li H.; Wen D.; Gu Z.; Gu Z. Colloidal Crystal Microneedle Patch for Glucose Monitoring. Nano Today 2020, 35, 100984 10.1016/j.nantod.2020.100984.

[ref133] Li X.; Lv J.; Zhao J.; Ling G.; Zhang P. Swellable Colorimetric Microneedles for Glucose Detection Based on Glucose Oxidase-like Gold Nanoparticles. Anal. Chim. Acta 2024, 1288, 342152 10.1016/j.aca.2023.342152. 38220286

[ref134] Wu T.; You X.; Chen Z. Hollow Microneedles on a Paper Fabricated by Standard Photolithography for the Screening Test of Prediabetes. Sensors 2022, 22 (11), 4253 10.3390/s22114253. 35684875 PMC9185271

[ref135] Dai Y.; Nolan J.; Madsen E.; Fratus M.; Lee J.; Zhang J.; Lim J.; Hong S.; Alam M. A.; Linnes J. C.; Lee H.; Lee C. H. Wearable Sensor Patch with Hydrogel Microneedles for In Situ Analysis of Interstitial Fluid. ACS Appl. Mater. Interfaces 2023, 15 (49), 56760–56773. 10.1021/acsami.3c12740. 38041570

[ref136] Yang B.; Fang X.; Kong J. Engineered Microneedles for Interstitial Fluid Cell-Free DNA Capture and Sensing Using Iontophoretic Dual-Extraction Wearable Patch. Adv. Funct. Mater. 2020, 30 (24), 2000591 10.1002/adfm.202000591.

[ref137] Zheng L.; Zhu D.; Xiao Y.; Zheng X.; Chen P. Microneedle Coupled Epidermal Sensor for Multiplexed Electrochemical Detection of Kidney Disease Biomarkers. Biosens. Bioelectron. 2023, 237, 115506 10.1016/j.bios.2023.115506. 37473548

[ref138] Tu J.; Min J.; Song Y.; Xu C.; Li J.; Moore J.; Hanson J.; Hu E.; Parimon T.; Wang T.-Y.; Davoodi E.; Chou T.-F.; Chen P.; Hsu J. J.; Rossiter H. B.; Gao W. A Wireless Patch for the Monitoring of C-Reactive Protein in Sweat. Nat. Biomed. Eng. 2023, 7 (10), 1293–1306. 10.1038/s41551-023-01059-5. 37349389 PMC10592261

[ref139] Enocsson H.; Karlsson J.; Li H.-Y.; Wu Y.; Kushner I.; Wetterö J.; Sjöwall C. The Complex Role of C-Reactive Protein in Systemic Lupus Erythematosus. J. Clin. Med. 2021, 10 (24), 5837 10.3390/jcm10245837. 34945133 PMC8708507

[ref140] Yang D.-H.; Yang S.-K.; Park S. H.; Lee H.-S.; Boo S.-J.; Park J.-H.; Na S. Y.; Jung K. W.; Kim K.-J.; Ye B. D.; Byeon J.-S.; Myung S.-J.; Kim J.-H. Usefulness of C-Reactive Protein as a Disease Activity Marker in Crohn’s Disease According to the Location of Disease. Gut Liver 2015, 9 (1), 80–86. 10.5009/gnl13424. 25170056 PMC4282861

[ref141] Sonani B.; Naganathan S.; Al-Dhahir M. A. Hypernatremia. In StatPearls; StatPearls Publishing: Treasure Island (FL), 2024.

[ref142] Sadiq N. M.; Naganathan S.; Badireddy M. Hypercalcemia. In StatPearls; StatPearls Publishing: Treasure Island (FL), 2024. 28613465

[ref143] Castro D.; Sharma S. Hypokalemia. In StatPearls; StatPearls Publishing: Treasure Island (FL), 2024.

[ref144] Sarnowski A.; Gama R. M.; Dawson A.; Mason H.; Banerjee D. Hyperkalemia in Chronic Kidney Disease: Links, Risks and Management. Int. J. Nephrol. Renov. Dis. 2022, 15, 215–228. 10.2147/IJNRD.S326464. PMC9356601 35942480

[ref145] Hill Gallant K. M.; Spiegel D. M. Calcium Balance in Chronic Kidney Disease. Curr. Osteoporos. Rep. 2017, 15 (3), 214–221. 10.1007/s11914-017-0368-x. 28474258 PMC5442193

[ref146] Borrelli S.; Provenzano M.; Gagliardi I.; Michael A.; Liberti M. E.; De Nicola L.; Conte G.; Garofalo C.; Andreucci M. Sodium Intake and Chronic Kidney Disease. Int. J. Mol. Sci. 2020, 21 (13), 4744 10.3390/ijms21134744. 32635265 PMC7369961

[ref147] Omar R.; Yuan M.; Wang J.; Sublaban M.; Saliba W.; Zheng Y.; Haick H. Self-Powered Freestanding Multifunctional Microneedle-Based Extended Gate Device for Personalized Health Monitoring. Sens. Actuators B Chem. 2024, 398, 134788 10.1016/j.snb.2023.134788. 38164440 PMC10652171

[ref148] Sprunger Y.; Capua L.; Ernst T.; Barraud S.; Locca D.; Ionescu A.; Saeidi A. pH Quantification in Human Dermal Interstitial Fluid Using Ultra-Thin SOI Silicon Nanowire ISFETs and a High-Sensitivity Constant-Current Approach. Biosensors 2023, 13 (10), 908 10.3390/bios13100908. 37887101 PMC10605508

[ref149] Enns G. M.; Cowan T. M. Glutathione as a Redox Biomarker in Mitochondrial Disease—Implications for Therapy. J. Clin. Med. 2017, 6 (5), 50 10.3390/jcm6050050. 28467362 PMC5447941

[ref150] Zhao J.; Lv J.; Ling G.; Zhang P. A Swellable Hydrogel Microneedle Based on Cerium-Metal Organic Frame Composite Nanozyme for Detection of Biomarkers. Int. J. Biol. Macromol. 2024, 254, 127745 10.1016/j.ijbiomac.2023.127745. 38287590

[ref151] Hoxha M.; Malaj V.; Zappacosta B. Health Economic Evaluations of Hemochromatosis Screening and Treatment: A Systematic Review. PharmacoEconomics - Open 2024, 8 (2), 147–170. 10.1007/s41669-023-00463-6. 38279979 PMC10884378

[ref152] Goud K. Y.; Moonla C.; Mishra R. K.; Yu C.; Narayan R.; Litvan I.; Wang J. Wearable Electrochemical Microneedle Sensor for Continuous Monitoring of Levodopa: Toward Parkinson Management. ACS Sens. 2019, 4 (8), 2196–2204. 10.1021/acssensors.9b01127. 31403773

[ref153] Han J. H.; Kim C. R.; Min C. H.; Kim M. J.; Kim S.-N.; Ji H. B.; Yoon S. B.; Lee C.; Choy Y. B. Microneedles Coated with Composites of Phenylboronic Acid-Containing Polymer and Carbon Nanotubes for Glucose Measurements in Interstitial Fluids. Biosens. Bioelectron. 2023, 238, 115571 10.1016/j.bios.2023.115571. 37562343

[ref154] Hee P. K. Biomarker Screening for Immunotherapy Response Evaluation Using Microneedle Patch in Patients With Allergic Rhinitis; Clinical trial registration NCT05922176; clinicaltrials.gov, 2023. https://clinicaltrials.gov/study/NCT05922176 (accessed 2024–01–01).

[ref155] Felner E. Glucose Measurement Using Microneedle Patches; Clinical trial registration NCT02682056; clinicaltrials.gov, 2019. https://clinicaltrials.gov/study/NCT02682056 (accessed 2024–01–01).

[ref156] Litvan I. Real-Time Levodopa Monitoring for Improved Management of Parkinson Disease; Clinical trial registration NCT04735627; clinicaltrials.gov, 2023. https://clinicaltrials.gov/study/NCT04735627 (accessed 2024–01–01).

[ref157] Imperial College London. Microneedle Sensing of Beta-Lactam Antibiotic Concentrations in Human Interstitial Fluid; Clinical trial registration NCT03847610; clinicaltrials.gov, 2020. https://clinicaltrials.gov/study/NCT03847610 (accessed 2024–01–01).

[ref158] Imperial College London. Closed-Loop Control of Penicillin Delivery Integrating Electrochemical Biosensor Technology; Clinical trial registration NCT04053140; clinicaltrials.gov, 2022. https://clinicaltrials.gov/study/NCT04053140 (accessed 2024–01–01).

[ref159] Friedel M.; Werbovetz B.; Drexelius A.; Watkins Z.; Bali A.; Plaxco K. W.; Heikenfeld J. Continuous Molecular Monitoring of Human Dermal Interstitial Fluid with Microneedle-Enabled Electrochemical Aptamer Sensors. Lab. Chip 2023, 23 (14), 3289–3299. 10.1039/D3LC00210A. 37395135 PMC11875127

[ref160] Cari Health Inc. Assessment of Methadone Dose Taken Using Electrochemistry; Clinical trial registration NCT05981573; clinicaltrials.gov, 2023. https://clinicaltrials.gov/study/NCT05981573 (accessed 2024–01–01).

[ref161] Imperial College London. Minimally-Invasive Realtime Assessment of Continuous Lactate in Exercise; Clinical trial registration NCT04238611; clinicaltrials.gov, 2021. https://clinicaltrials.gov/study/NCT04238611 (accessed 2024–01–01).

[ref162] Mordor Intelligence. Microneedle Drug Delivery Systems Market Size & Share Analysis - Industry Research Report - Growth Trends. https://www.mordorintelligence.com/industry-reports/microneedle-drug-delivery-systems-market (accessed 2023-09–07).

[ref163] Future Market Insights. Microneedle Drug Delivery Systems Market. https://www.futuremarketinsights.com/reports/microneedle-drug-delivery-systems-market (accessed 2023-09–07).

[ref164] https://www.emergenresearch.com, E. R. Microneedling Market Trend | Industry Forecast 2021–2030. https://www.emergenresearch.com/industry-report/microneedling-market (accessed 2024-01–18).

[ref165] Microneedle Drug Delivery Systems Market Size Report, 2030. https://www.grandviewresearch.com/industry-analysis/microneedle-drug-delivery-systems-market-report (accessed 2024-01–18).

[ref166] Microneedling Market Size | Share | Trend | Revenue Report by 2030. https://www.emergenresearch.com/press-release/global-microneedling-market (accessed 2024-01–18).

[ref167] Research and Markets. Global Microneedle Market 2021–2026 - Research and Markets. https://www.researchandmarkets.com/reports/5393472/global-microneedle-market-2021-2026 (accessed 2023-09–07).

[ref168] Point Of Care Diagnostics Market Size & Share Report, 2030. https://www.grandviewresearch.com/industry-analysis/point-of-care-poc-diagnostics-industry (accessed 2024-01–18).

[ref169] Point of Care Diagnostics Market Size, Share & Trends [2030]. https://www.fortunebusinessinsights.com/industry-reports/point-of-care-diagnostics-market-101072 (accessed 2024-01–18).

[ref170] Raphas Co. First, Core Technology. http://www.raphas.com/en/tech/core (accessed 2023-08–03).

[ref171] MITI Systems. Eye Patch | Soluble microneedle patch, Dissolving microneedle patch, eye patch, wrinkle patch. http://www.mitisystems.com/en/eye_patch.html (accessed 2023-08–25).

[ref172] CosMED Pharmaceutical Co., Ltd. Microneedles | CosMED Pharmaceutical Co., Ltd. https://cosmed-pharm.co.jp/en/innovation/ (accessed 2023-08–03).

[ref173] Think-Lands Co., Ltd. Micro Needle Business. https://www.think-lands.co.jp/ (accessed 2023-08–03).

[ref174] Micropoint Technologies. Micropoint Patch. Micropoint Technologies. https://micropoint-tech.com/products/micropoint-patch/ (accessed 2023-08–03).

[ref175] Vaxess Technologies. MIMIX^TM^ Therapies. https://www.vaxess.com/mimix-therapies (accessed 2023-08–03).

[ref176] LTS Lohmann Therapie-Systeme AG. LTS Micro Array Patches. LTS. https://www.ltslohmann.com/en/our-technologies/map/ (accessed 2023-08–03).

[ref177] Innoture Medical Technology Limited. Our Platform Technology. https://innoture.co/our-platform-technology/ (accessed 2023-08–03).

[ref178] Avcil M.; Çelik A. Microneedles in Drug Delivery: Progress and Challenges. Micromachines 2021, 12 (11), 1321 10.3390/mi12111321. 34832733 PMC8623547

